# CCR2^+^ macrophages are required for exercise-induced cardiac remodeling

**DOI:** 10.3389/fphar.2026.1844427

**Published:** 2026-06-25

**Authors:** Gianni Bonnici, Tristan Cobb, McKenna Burns, Camille Daigle, Maura Sticco-Ivins, Elizabeth Hardy, Julie Lang, Hao Dun, Samantha L. Nelson, Brisa Peña, Sue Gu, Benjamin J. Kopecky

**Affiliations:** 1 Division of Cardiology, Department of Medicine, University of Colorado Anschutz, Aurora, CO, United States; 2 Department of Biomedical Engineering, University of Colorado Anschutz, Aurora, CO, United States; 3 Division of Pulmonary, Allergy, and Critical Care Medicine, Department of Medicine, University of Colorado Anschutz, Aurora, CO, United States; 4 Department of Immunology, University of Colorado Anschutz, Aurora, CO, United States

**Keywords:** cardiac remodeling, exercise, macrophage, reprogramming, resident macrophages

## Abstract

**Background:**

Cardiovascular diseases (CVDs) remain the leading cause of mortality worldwide, with exercise emerging as a potential non-pharmacological strategy to reduce adverse outcomes. Prior studies examining macrophage responses to exercise primarily focused on recruited C-C motif chemokine receptor 2 (CCR2^+^) monocytes and macrophages in blood, adipose tissue, and skeletal muscle, but the physiological role of macrophages within the heart remains poorly defined.

**Objectives:**

To determine the role of CCR2^+^ macrophages in exercise-induced cardiac remodeling in both male and female mice.

**Methods:**

We validated a voluntary exercise wheel-running model with diphtheria toxin mediated depletion of CCR2^+^ macrophages. Cardiac remodeling was assessed using morphometric indices, echocardiography, and tissue analysis.

**Results:**

Echocardiography revealed voluntary exercise increased left ventricle (LV) mass and wall thickness while preserving cardiac function compared to sedentary conditions. Exercise increased morphometric heart, LV, and right ventricle (RV) mass as well as Fulton index. Cardiomyocyte cross-sectional area was also increased in both the LV and RV of voluntary exercise mice. Cardiac remodeling occurred in both sexes, with sex-specific differences in the LV. Immunofluorescence quantification of interstitial CD68^+^ cardiac macrophages revealed increased CCR2^+^ macrophages and increased CCR2^+^ to CCR2^−^ macrophage ratios in both LV and RV of voluntary exercise mice. Notably, depletion of CCR2^+^ macrophages prevented exercise-induced cardiac hypertrophy in echocardiography, morphometric, and cardiomyocyte cross-sectional area measures. CCR2^+^ depletion also decreased cardiomyocyte stiffness, regardless of condition, while no changes in fibrosis were observed.

**Conclusion:**

Together, these findings identify CCR2^+^ macrophages as required for exercise-induced cardiac remodeling and provide new insight into immune-cardiac interactions underlying adaptation to exercise.

## 1 Introduction

Cardiovascular diseases (CVDs) remain the leading cause of mortality worldwide, with heart disease continuing to be the primary cause of death in the United States (Ahmad et al., 2024; Di Cesare et al., 2024). CVD encompasses a broad spectrum of conditions, each involving complex interactions between cardiac tissue and the immune system (Schwinger, 2021; Shimizu and Minamino, 2016). Resident cardiac macrophages have emerged as central regulators of both the progression and resolution of numerous cardiac disorders, including hypertension (Kopecky and Lavine, 2022), myocarditis (Ma et al., 2024), myocardial infarction (Bajpai et al., 2019), heart failure with preserved ejection fraction (Raman et al., 2025), and heart transplant outcomes (Kopecky et al., 2022; Kopecky et al., 2020).

Exercise has gained recognition as a promising non-pharmacological intervention capable of improving cardiovascular health (Kunutsor and Laukkanen, 2024). Growing evidence suggests that the immune system may play a pivotal role in mediating exercise-induced cardiovascular remodeling (Dixit et al., 2023; Walzik et al., 2026). While immune cell contributions to pathological cardiac remodeling are increasingly well-defined, their roles in exercise-induced *physiological* remodeling remain largely unexplored. Notably, excessive or prolonged endurance exercise has been linked to irreversible cardiac injury, raising critical questions about the dose-dependent effects of physical activity on the heart (O'Keefe et al., 2012; Franklin et al., 2020; Guarnieri et al., 2025). Because exercise can act both as a disease-modifying therapy and as a physiological stressor capable of inducing reversible cardiac hypertrophy, understanding how cardiac immune cells respond to exercise is essential for informing personalized exercise prescriptions across diverse clinical contexts.

Resident cardiac macrophages are key players in these processes and can be broadly divided into C-C motif chemokine receptor 2 negative (CCR2^-^) and CCR2^+^ subsets. CCR2^-^ macrophages, derived from embryonic progenitors, self-renew locally and contribute to coronary vessel maturation, myocardial growth, and neonatal heart regeneration (Bajpai et al., 2019; Bajpai et al., 2018; Lavine et al., 2014; Li et al., 2016; Patel et al., 2018; Yerra and Advani, 2022). In contrast, CCR2^+^ macrophages arise from adult hematopoietic progenitors, depend on monocyte recruitment, and mediate inflammatory cascades following cardiomyocyte injury, often promoting adverse remodeling (Bajpai et al., 2019; Lavine et al., 2014; Dick et al., 2019; Epelman et al., 2014; Leid et al., 2016).

Although the balance and function of CCR2^-^ and CCR2^+^ macrophages have been extensively investigated in models of cardiac disease (Bajpai et al., 2019; Patel et al., 2018; Revelo et al., 2021; Wen et al., 2026; Wong et al., 2021), very few studies have examined how these populations respond in during exercise-induced physiologic cardiac remodeling. Prior work has predominantly focused on monocyte dynamics in blood, adipose tissue, and skeletal muscle rather than in cardiac tissue itself (Blanks et al., 2020; Blanks et al., 2022; Kawanishi et al., 2010; Walton et al., 2019; Okutsu et al., 2008). As a result, the direct contributions of cardiac macrophage subsets to exercise-induced cardiac remodeling remain an open and clinically important question.

Herein, we implemented a voluntary exercise model to study exercise-induced cardiac hypertrophy using echocardiography, morphometric assessment, and tissue-level analyses. We quantified CCR2^+^ and CCR2^-^ macrophage populations in both the left and right ventricles to define exercise-driven shifts in macrophage composition. Through targeted ablation of CCR2^+^ macrophages, we identified a previously unrecognized requirement for CCR2^+^ macrophages in exercise-induced cardiac hypertrophy. Moreover, we hypothesize that sex differences further shape both immune and remodeling responses. Together, these findings reveal a critical role for CCR2^+^ macrophages in orchestrating physiologic cardiac adaptation to exercise.

## 2 Materials and methods

### 2.1 Animals

Mice were bred and maintained at the University of Colorado Anschutz School of Medicine with free access to water and a standard chow diet on a 14:10 h light-dark cycle. All animal procedures were approved by the Institutional Animal Care and Use Committee (IACUC) #01386. Mouse strains used included C57BL/6J (Jax#000664), CX3CR1^GFP/+^ CCR2^RFP/+^ (Jax#032127) (Jung et al., 2000; Saederup et al., 2010), and CCR2^DTR/+^ (Hohl et al., 2009). Male and female mice aged 8–10 weeks were randomly assigned to sedentary control or voluntary exercise (VE) groups with three cohorts in total. Cohort 1 consisted of C57BL/6J and ran for 15 weeks. Cohort 2 consisted of CX3CR1^GFP/+^ CCR2^RFP/+^ (dual reporter (DR) mice) and ran for 23–25 weeks. Cohort 3 consisted of CCR2^DTR/+^ and CCR2^+/+^ littermate controls and ran for 22 weeks. VE mice were individually housed with a running wheel [wireless low-profile mouse running wheel (Med Associates, Vermont, United States, MED Associates ENV-047)] and able to run voluntarily. Running data was tracked using Wireless Device USB Hub (MED Associates, DIG-807). CCR2^DTR/+^ and CCR2^+/+^ mice were given 200 ng intraperitoneal injections of diphtheria toxin (1 μg/mL; Sigma-Aldrich Cat #D0564) on three consecutive days then twice weekly thereafter. In CCR2^DTR/+^ mice, CCR2-expressing cells can be selectively depleted following administration of diphtheria toxin. Throughout the manuscript, CCR2^DTR/+^ mice are referred to as DTR^+^, whereas mice lacking the DTR allele are referred to as DTR^−^ controls. Wild-type C57BL/6J, CCR2^+/+^, and CX3CR1^GFP/+^ CCR2^RFP/+^ “Dual Reporter” mice that did not carry the DTR allele were pooled and analyzed together as DTR^−^ controls because these genotypes do not permit diphtheria toxin-mediated CCR2^+^ cell depletion.

### 2.2 Wheel running analysis

Wheel-running data were exported from Wheel Manager software (Version 2.04, MED Associates) as revolutions per minute and processed using a custom MATLAB script (https://github.com/g6bonnici/ExerciseWheelTracking). A day was included in the analysis only if at least 23 h of data were recorded, with the active period defined as the 10-h dark cycle (lights off) and the inactive period as the 14-h light cycle (lights on). Rarely, loss of transmission from the Wireless Device USB Hub occurred; in these cases, active and inactive periods were normalized separately based on their respective recorded durations to account for data gaps. Running distance was calculated from wheel revolutions using the wheel radius (6.0198 cm) to determine circumference. Total distance run was calculated by taking the area under the curve using GraphPad Prism (Version 10.6.1, Inc., San Diego, California) for the average distance per day during the first 15 weeks.

### 2.3 Echocardiogram

Echocardiogram methodology was adapted from previously published protocols (Rubino et al., 2023; Eggertsen et al., 2025). Briefly, transthoracic echocardiographic and Doppler analysis were performed using the VisualSonics Vevo F2 imaging system. Mice were anesthetized with 2.5% isoflurane and hair was removed from the chest with depilatory cream. Vitals and body temperature (maintained at 37 °C) were monitored with a heated platform embedded with electrodes. Isoflurane was then lowered to and maintained at 1.5% throughout the procedure and across subjects. Parasternal long axis (PSLAX) and parasternal short axis (SAX) views of the left ventricle (LV) were obtained; with SAX, two-dimensional views used to acquire M-mode images. Anterior and posterior LV wall thickness and internal diameters were measured in systole and diastole using the M-mode images to calculate percent LV ejection fraction (EF%). Mitral inflow Color Doppler and myocardial tissue movement of the mitral annulus were captured via modified apical four-chamber view. Ratio of early (E) to active (A) filling waves of blood flow through the mitral valve (E/A), along with ratio of myocardial early (E′) to active (A′) annular tissue velocity (E’/A′), were used to assess cardiac diastolic function. LV mass was calculated through the Troy formula (Troy et al., 1972; Foppa et al., 2005):

$$LV\;mass\;\left(mg\right)=1.053\;x\;\left[{\left(LVID;d+LVPW;d+IVS;d\right)}^{3}-{LVID;d}^{3}\right]$$

LVID; d = Left Ventricular Internal Diameter in Diastole. LVPW; d = Posterior Wall Thickness in Diastole. IVS; d = Interventricular Septum Thickness in Diastole. All echocardiographic measurements and analyses were averaged from at least four cardiac cycles. Analyses were performed in a blinded manner by an imaging specialist.

### 2.4 Tissue analysis

Hearts were flushed with 5 mL ice cold phosphate buffered saline (PBS) with left and right ventricle separation. Digital calipers were used to assess tibia length from the tibial head (exposed after the patellar tendon is severed) to the space within the ankle exposed after severing the Achilles tendon. Hearts were then either fixed overnight in 4% paraformaldehyde (PFA) at 4 °C or fresh-frozen embedded in O.C.T. (Fisher Healthcare Tissue Plus O.C.T. Compound Cat #4585). Hearts fixed in 4% PFA were rinsed with PBS thrice and manually divided with a razorblade. Sections were then either infiltrated with 30% sucrose (in PBS) overnight at 4 °C or sent to the University of Colorado Anschutz Gates Institute Histology Core for formalin-fixed paraffin-embedding (FFPE) and sectioning (4 µm thick). Hearts in 30% sucrose were O.C.T embedded, cut into 10 µm slices using a Leica Cryostat, and frozen at −80 °C.

#### 2.4.1 Immunofluorescence

O.C.T sections were thawed for 10 min at room temperature, washed in PBS, and blocked with 10% bovine serum albumin (BSA) in TBS-T (0.05% Tween-20) for 1 h at room temperature. Sections were then incubated overnight at 4 °C in a humidified chamber with either the following primary antibodies diluted in blocking buffer: rabbit anti-CD68 (1:250, Cell Signaling Technology Cat# 97778), chicken anti-GFP (1:2000, Abcam Cat# 13970), and rat anti-RFP (1:500, ChromoTek Cat# 5F8-20) or rabbit anti-RFP (1:500, Rockland Cat# 600-401-379), chicken anti-GFP (1:2000, Abcam Cat# 13970), and rat anti-Ki67 (1:200, eBioscience Cat# 14-5698-82). Following primary incubation, sections were washed with TBS-T and stained for 1 h at room temperature, protected from light, with either the following secondary antibodies, each diluted 1:500 in blocking buffer: goat anti-rabbit Alexa Fluor 647 (Invitrogen Cat# A21245), goat anti-chicken Alexa Fluor 488 (Invitrogen Cat# A11309), and goat anti-rat Alexa Fluor 555 (Invitrogen Cat# A21434) or goat anti-rabbit Alexa Fluor 555 (Invitrogen Cat# A21428), goat anti-chicken Alexa Fluor 488 (Invitrogen Cat# A11309), and goat anti-rat Alexa Fluor 647 (Invitrogen Cat# A21247). Nuclei were counterstained with DAPI (Invitrogen Cat# D3571) for 5 min at room temperature. After final washes, slides were mounted using Prolong Diamond Antifade Mountant (Invitrogen Cat# P36961) and coverslipped. Images were acquired at 20x using an Olympus VS200 slide scanner (Olympus Corporation, Tokyo, Japan) under consistent exposure settings for all samples. Whole tissue sections were blindly and manually quantified for CCR2^+^ and CCR2^−^ cardiac macrophages which were identified by co-expression of CX3CR1-GFP and/or CCR2-RFP reporters together with the macrophage marker CD68. CCR2^−^ macrophages were defined as CX3CR1-GFP^+^ CCR2-RFP^−^ CD68^+^ cells, whereas CCR2^+^ macrophages were defined as CX3CR1-GFP^+^ CCR2-RFP^+^ CD68^+^ cells. Quantification was restricted to cells localized within the myocardial interstitium (using autofluorescence of cardiac tissue to define boundaries) and excluded intravascular cells when morphologically identifiable.

#### 2.4.2 Wheat germ agglutinin (WGA)

O.C.T sections were thawed for 10 min at room temperature, washed in Hanks’ Balanced Salt Solution (HBSS), and blocked with 10% bovine serum albumin (BSA) in HBSS for 30 min at room temperature. Sections were then incubated at room temperature for 30 min with WGA-Alexa Fluor 488 (10 ug/mL, Invitrogen Cat #W11261). Following primary incubation, sections were washed with HBSS, and nuclei were counterstained with DAPI (Invitrogen Cat# D3571) for 5 min at room temperature. After final washes, slides were mounted using Prolong Diamond Antifade Mountant (Invitrogen Cat# P36961) and coverslipped. Images were acquired at 20x using an Olympus VS200 slide scanner under consistent exposure settings for all samples. Both left and right ventricle sections were analyzed by randomly selecting 250 μm × 250 µm areas of cross-sectionally oriented cardiomyocytes in QuPath v0.6.0. Each cross-sectional area was hand traced to quantify cardiomyocyte area (Supplementary Figure 11). Data points were represented by averaging all cardiomyocyte cross-sectional areas within a section.

#### 2.4.3 Picrosirius red (PSR)

FFPE tissue was stained for PSR (Abcam Cat# ab150681) per manufacturer’s protocol. Images were acquired at 20x using an Olympus VS200 slide scanner under consistent exposure settings for all samples. Each sample was analyzed using a custom Fiji Macro (https://github.com/CompSciBio/Macros/) to automatically color threshold for slide background (Hue: 0–255; Saturation: 0–35; Brightness: 0–255), collagen-negative tissue (Hue: 0–39; Saturation: 35–255; Brightness: 0–255), and collagen-positive staining (Hue: 220–255; Saturation: 60–255; Brightness: 0–255). These values for thresholding were determined via a manual subset and visually confirmed with each masking layer. Fibrosis (%) was determined as the fraction of collagen-positive pixels relative to total tissue pixels (total pixels minus slide background).

#### 2.4.4 Atomic force microscopy (AFM)

AFM, in force spectroscopy mode, was used to quantify the nanomechanical properties of fresh-frozen cardiac tissue sections (6 µm thickness). Measurements were performed using a NanoWizard® 4a system (JPK Instruments) equipped with MLCT cantilevers (Bruker). This methodology was adapted from previously published protocols for AFM analysis of frozen biological tissues and optimized for our instrumentation (Kuwabara et al., 2022; Lachaize et al., 2021; Mueller et al., 2021; Pena et al., 2022a; Pena et al., 2022b; Pena et al., 2025; Travers et al., 2021; Zanetti et al., 2024). Briefly, frozen cardiac sections were allowed to thaw at room temperature and subsequently immersed in phosphate-buffered saline (PBS) for 15 min to remove residual OCT embedding compound. During AFM measurements, tissue sections were maintained in PBS supplemented with 1x protease inhibitor cocktail (HALT, Thermo Fisher Scientific Cat#78442) to preserve tissue integrity. Cantilever spring constants were calibrated prior to each experiment using the thermal tune method and ranged between 0.01 and 0.02 N/m. Force distance curves were acquired with a maximum applied force limited to 1.5 nN to minimize tissue damage, using a Z-piezo travel of 5 µm. For each sample, at least 50 indentations, in cardiomyocyte cells, were performed across five distinct, randomly selected cardiomyocyte rich regions within the tissue section. At least two tissues were analyzed per group, and all measurements, including data analysis and data collection, were conducted in a blinded manner. All experimental parameters including probe type, indentation force, approach speed, environmental conditions, and sample preparation, were kept constant across all groups. The apparent elastic modulus was calculated by fitting the force indentation curves to the Hertz contact model using JPK analysis software.

### 2.5 Spectral flow cytometry

#### 2.5.1 Tissue preparation

Heart single cell suspensions were achieved by flushing with ice cold PBS and sectioning ∼40 mg from apex of heart which was manually digested by mincing. Heart was then enzymatically digested in DMEM with Collagenase IV (Sigma C5138) 4500 U/mL in DMEM, Hyaluronidase 1 (Sigma H3506) 2400 U/mL in DMEM, and DNAse I (Sigma D4527-40KU) 6000 U/mL in DMEM for 40 min at 37 °C with shaking. On completion, digestion was quenched with the addition of 6 mL HBSS with 2% FBS and 0.2% BSA, then the cell solution was passed through a 70 μm cell strainer. After centrifugation at 400 *g* for 6 min at 4 °C, samples were resuspended in 1 mL ACK Lysis buffer (Thermo-Fisher Scientific) for 5 min at room temperature for red blood cell lysis before quenching with 9 mL DMEM. After centrifugation at 400 *g* for 10 min at 4 °C, cells were resuspended in 150 µL of flow cell media (RPMI, 5% BSA, 3.33% DNase I) and transferred to 96-well round bottom plate.

#### 2.5.2 Staining procedure

Samples were centrifuged at 652 *g* for 3 min at 4 °C and resuspended and stained in 100uL L/D blue diluted in PBS for 15 min at room temperature on shaker. Samples were centrifuged at 652 *g* for 3 min at 4 °C and stained for 15 min at 4 °C on shaker in the dark according to antibody mix in Table 1 diluted in staining buffer (PBS, 1% BSA, 0.1% sodium azide). Samples were then centrifuged at 652 *g* for 3 min at 4 °C, washed with staining buffer, centrifuged at 652 *g* for 3 min at 4 °C, and resuspended in final volumes of 250 µL for heart.

**TABLE 1:** Spectral flow cytometry antibody panel.

Antibody	Clone	Fluor	Company	Dilution
Live dead	​	L/D blue	Invitrogen	2000x
*Antibody staining mix*				
mCD45	30-F11	BUV395	Invitrogen	225x
CD11c	K418	BUV615	BD bioscience	112.5x
CD24	M1/69	BUV737	BD bioscience	562.5x
H-2 class I pan	M1-42	BUV805	BD bioscience	225x
CD64	W18349C	BV421	BioLegend	112.5x
CD31	390	PacB	BioLegend	562.5x
CD172a SIRPa	P84	BV480	BioLegend	225x
Ly6C	HK1.4	BV605	BioLegend	225x
Ly6G	1A8	BV785	BioLegend	225x
CCR2	Y15-488.rMAb	BD RY586	BD bioscience	112.5x
CD86	GL-1	SparkBlue plus 550	BioLegend	225x
I-A/I-E	M5/114.15.2	PerCP/Fire 806	BioLegend	562.5x
CD80	16-10A1	PeDazzle594	BioLegend	562.5x
F4/80	BM8	PEFire640	BioLegend	225x
NK1.1 (C57Bl/6)	PK136	PeFire 700	BioLegend	225x
CD40	3/23	PeCY7	BioLegend	562.5x
Feeder	MEF-SK4	APC	Miltenyi	112.5x
CD3	17A2	APC	BioLegend	112.5x
B220	RA3-6B2	APC	BioLegend	112.5x
CD11b	M1/70	SN685	BioLegend	225x
CD169	Ser4	APCFire750	Invitrogen	225x
CD16/CD32	2.4G2	Fc block	BD bioscience	225x

#### 2.5.3 Spectral unmixing

The 5-Laser Aurora spectral cytometer (Aurora 5L, Cytek Biosciences, United States) was used to acquire stained cells. Unstained controls for each tissue type were used as reference controls for spectral unmixing in each experiment. Autofluorescence extraction was applied to each data set during spectral unmixing. Where possible, single-stained references were generated using cells from bone marrow tissues, otherwise beads were used. Bone marrow was also used as a positive control to ensure adequate staining of antibody mix from Table 1.

The data acquired was analyzed using FlowJo (v10.8, BD Biosciences, United States).

### 2.6 qPCR

Total RNA was isolated from fresh frozen right ventricle tissue using TRIzol Reagent (Thermo Fisher Scientific) according to the manufacturer’s instructions in a standard phenol/chloroform extraction (Burns et al., 2024). RNA concentration and purity were assessed via NanoDrop and 1,000 ng of RNA was reverse transcribed using the BioRad iScript (https://www.bio-rad.com/de-de/product/iscript-cdna-synthesis-kit?ID=M87EWZESH. iScript™ cDNA Synthesis Kit | Bio-Rad) kit. Quantitative PCR (qPCR) was performed on the Applied Biosystems QuantStudio 5 Real-Time PCR System. Samples were run in triplicate and normalized to the housekeeping gene 18S rRNA through calculation of ΔCt values [ΔCt = Ct (sample) − Ct (18S rRNA)]. Transcript expression levels as fold change were calculated from 2^−ΔΔCt^ normalized to sedentary. The following primer sequences were used: Myh7 (Fwd GCT​GGA​AGA​TGA​GTG​CTC​AGA​G) (Rev TCC​AAA​CCA​GCC​ATC​TCC​TCT​G); Myh6 (Fwd GCT​GGA​AGA​TGA​GTG​CTC​AGA​G) (Rev CCA​GCC​ATC​TCC​TCT​GTT​AGG​T); NPPB (Fwd TCC​TAG​CCA​GTC​TCC​AGA​GCA​A) (Rev GGT​CCT​TCA​AGA​GCT​GTC​TCT​G); NPPA (Fwd TAC​AGT​GCG​GTG​TCC​AAC​ACA​G) (Rev TGC​TTC​CTC​AGT​CTG​CTC​ACT​C).

### 2.7 Statistics

Statistical analyses were performed using GraphPad Prism (Version 10.6.1, Inc., San Diego, California). All results are shown as mean ± SEM. For 2-group comparisons, unpaired Student’s t-test for normally distributed variables and Mann-Whitney U test described non-normally distributed variables. One-way analysis of variance (ANOVA) with Dunnett’s multiple-comparison test was used to compare 3 groups with a single factor. Two-way ANOVA and two-way repeated measures (RM) ANOVA with Greisser-Greenhouse correction were used for comparisons involving 2 factors. Three-way RM ANOVA were used for comparisons involving 3 factors. Sidak’s multiple-comparisons test was used for 2 and 3 factor *post hoc* comparisons. Differences were considered significant when p < 0.05 (*), <0.01 (**) <0.001 (***), <0.0001 (****). Non-significant trends were shown between 0.05 < p < 0.11 with numerical p values.

## 3 Results

### 3.1 Prolonged voluntary exercise was sufficient to induce cardiac hypertrophy with sex-specific effects

**FIGURE 1:**
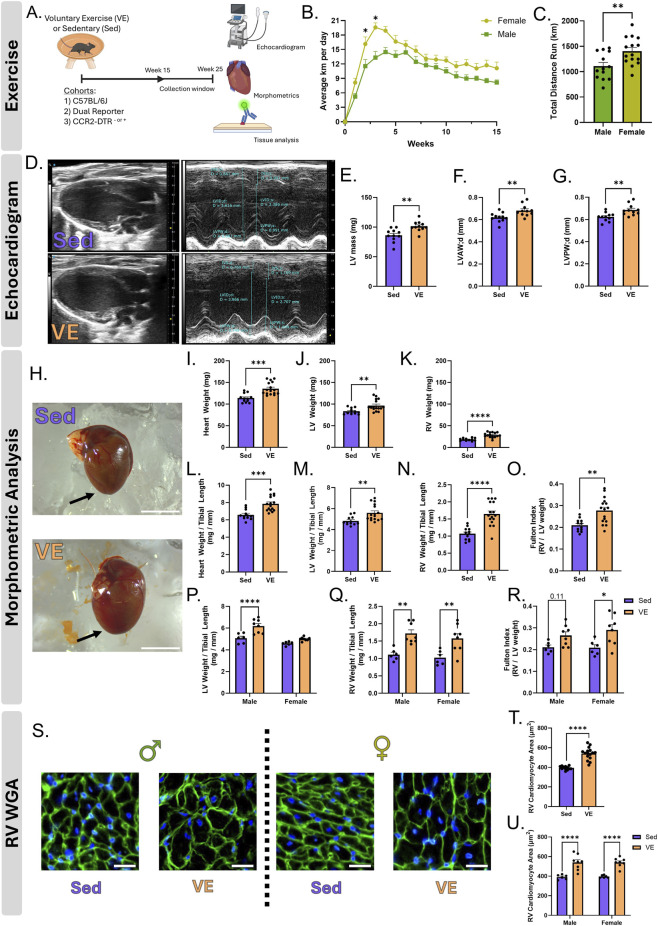
Prolonged voluntary exercise induces cardiac hypertrophy. **(A)** Experimental design for voluntary exercise (VE) and sedentary (Sed) groups. **(B)** Average distance run per day of male (n = 13) and female (n = 15) VE for first 15 weeks *p < 0.05 female vs. male. Two-way repeated measures ANOVA with Sidak’s multiple comparison. **(C)** Total distance ran over 15 weeks. **(D)** Representative images of left ventricle (LV) echocardiogram parasternal long axis (left) and m-mode (right) of female sedentary and VE. Echocardiogram measurements of **(E)** LV mass, **(F)** LVAW; d, LV diastolic anterior wall thickness, and **(G)** LVPW; d, LV diastolic posterior wall thickness; n = 11/group. **(H)** Representative gross heart image of sedentary and VE. Arrow depicts differences in heart apex cardiac remodeling. Scale bar = 5 mm. Unnormalized weight of **(I)** heart, **(J)** LV, and **(K)** right ventricle (RV). Tibia length normalized weight of **(L)** heart, **(M)** LV, and **(N)** RV as well as **(O)** Fulton index of sedentary and VE mice. Sex split tibial length normalized weights of **(P)** LV, **(Q)** RV, and **(R)** Fulton index. **(S)** Representative RV images of wheat germ agglutinin (WGA; green) immunofluorescence of sedentary and VE split by sex. Scale bar = 20 µm. RV cardiomyocyte cross-sectional area of **(T)** combined and **(U)** split male and female; n = 12–16/group with equal splits of male and females. Data represented as mean ± SEM. **(C,E–G,I,K,L,N,T)** Unpaired Student’s t-test; **(J,K)** Mann-Whitney U test; **(P,Q,U)** Two-way ANOVA with Sidak’s multiple comparison. *p < 0.05, **p < 0.01, ***p < 0.001, ****p < 0.001. ns = nonsignificant. [Created in BioRender. Bonnici, G. (2026) https://BioRender.com/a4om53c], [SF29UCH9VJ], [BioRender].

We first validated that our model of voluntary exercise (VE) could induce cardiac remodeling. Due to logistic restraints on wheel availability, to adequately power sex-specific analyses, and to maintain consistent control groups across cohorts, we aggregated groups when possible. All mice ran for at least 15 weeks followed by echocardiography, morphometrics, and tissue analysis (Figure 1A). Females ran farther than males in week 2, week 3, and overall (Figures 1B,C), with consistent activity observed during the 10-h active period across cohorts (Supplementary Figure 1A).

Echocardiography revealed significant increases in left ventricular (LV) mass (Figures 1D,E), LV anterior wall thickness in diastole (LVAW; d) (Figure 1F), and LV posterior wall thickness during diastole (LVPW; d) (Figure 1G) of VE groups compared to sedentary. There were no other significant echocardiographic changes (Supplementary Figures 1F–T). Because measurements such as E/E′ are known to increase with age, and as mice in this study were studied up to an age of 35 weeks (8 months), the observed values remain within acceptable age-related diastolic ranges (Supplementary Figures 1, 2, 7, 8) (de Lucia et al., 2019).

Cohorts two and three were used for morphometric and cardiomyocyte cross-sectional area (CSA) analysis as tibial lengths and ventricle separation were not performed in Cohort 1. Representative gross heart morphology demonstrates a larger heart in VE than in sedentary conditions (Figure 1H). Total heart weight (Figure 1I), LV weight (Figure 1J), and RV weight (Figure 1K) increased in VE and persisted through normalization to tibial length (Figures 1L–N) compared to sedentary groups. VE mice had a significant increase in Fulton index (RV weight/LV weight [LV free wall and septum]) (Figure 1O), suggesting a disproportionate RV > LV remodeling with exercise. When stratified by sex, increased LV weight was observed only in male VE mice (Figure 1P), whereas RV weight increased with VE in both sexes (Figure 1Q). Only females had a significant rise in Fulton index, as males demonstrated proportional increases in both RV and LV weight (Figure 1R).

To assess whether the increase in heart weight was due to cardiomyocyte hypertrophy, we performed WGA staining to calculate cardiomyocyte CSA. Representative images of male and female RV (Figure 1S) and LV (Supplementary Figure 1U) are shown. VE mice had a significant increase in both RV (Figure 1T) and LV (Supplementary Figure 1V) cardiomyocyte CSA. In sex-specific analyses, RV CSA was significantly increased in both male and female VE compared to sedentary controls (Figure 1U). Consistent with gross weights, male, but not female VE, displayed increased LV cardiomyocyte CSA (Supplementary Figure 1W).

In sum, echocardiography demonstrated LV remodeling in VE mice compared to controls, accompanied by increases in gross ventricular mass and cardiomyocyte CSA in both the LV and RV. When stratified by sex, only male VE mice had increased LV mass and CSA, whereas RV mass and CSA increased in both male and female VE mice compared with controls. *These findings support VE-induced cardiac remodeling in both sexes, with sex-specific differences in the LV.*

### 3.2 Cardiac macrophage differed in response to voluntary exercise

We next sought to determine whether exercise modulated the cardiac immune response. We noted that VE mice had larger spleens than sedentary controls (Supplementary Figures 1C,D). Given prior evidence linking cardiac macrophages and splenic immune reservoirs to adverse cardiac remodeling (Hiraiwa et al., 2022a), we analyzed cardiac macrophage composition in Cohort 2 mice using “Dual Reporter” mice.

**FIGURE 2:**
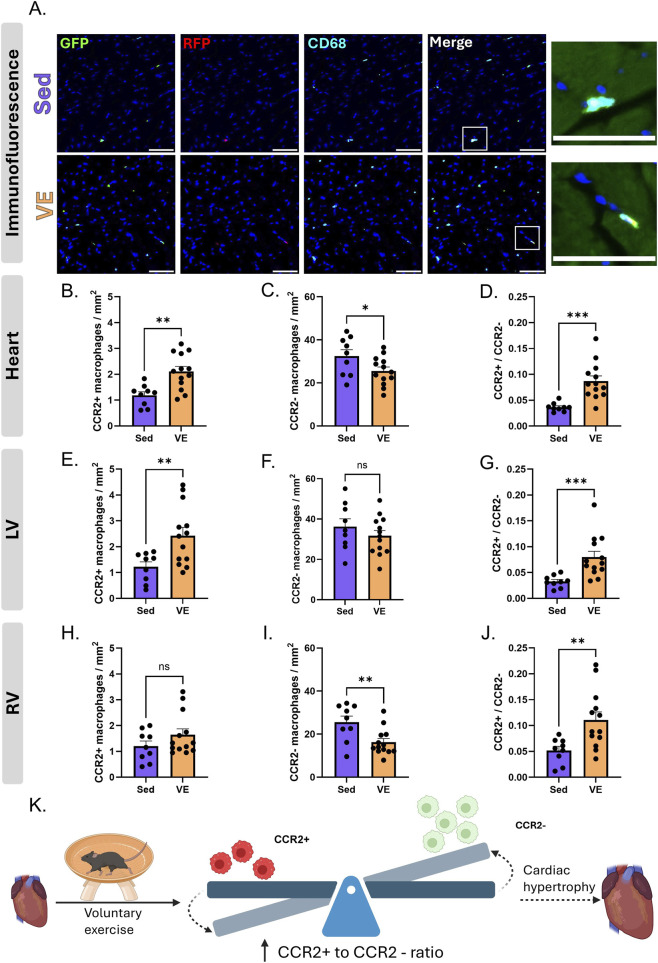
Voluntary exercise modulates cardiac CCR2^+^ and CCR2^-^ macrophage composition. **(A)** Representative low- and high-magnification immunofluorescence images demonstrating DAPI (blue), CX3CR1-GFP (green), CCR2-RFP (red), and CD68 (cyan) in sedentary and VE myocardium. Higher magnification images highlight interstitial localization and CD68 co-localization of CCR2^+^ and CCR2^−^ macrophages. Scale bar = 50 µm. Quantification of **(B)** heart (LV and RV combined) CCR2^+^ macrophages, **(C)** CCR2^-^ macrophages, and **(D)** ratio of CCR2^+^ to CCR2^-^ macrophages. Quantification of LV **(E)** CCR2^+^ macrophages, **(F)** CCR2^-^ macrophages, and **(G)** ratio of CCR2^+^ to CCR2^-^ macrophages. Quantification of RV **(H)** CCR2^+^ macrophages, **(I)** CCR2^-^ macrophages, and **(J)** ratio of CCR2^+^ to CCR2^-^ macrophages; n = 9–13/group. **(K)** Hypothesis schematic of macrophage role in VE induced cardiac hypertrophy. CX3CR1 = C-X3-C motif chemokine receptor 1. GFP = green fluorescent protein. CCR2 = C-C motif chemokine receptor 2. RFP = red fluorescent protein. Data represented as mean ± SEM. **(B–F,I,J)** Unpaired Student’s t-test; **(G,H)** Mann-Whitney U test. *p < 0.05, **p < 0.01, ***p < 0.001. Abbreviations as in Figure 1. [Created in BioRender. Bonnici, G. (2026) https://BioRender.com/5di380o], [KP29UCH448}], [BioRender].

Manual quantification of immunofluorescence was performed to identify interstitial cardiac macrophages using reporter expression together with the macrophage marker CD68 (Figure 2A). CCR2^−^ macrophages were defined as CX3CR1-GFP^+^ CCR2-RFP^−^ CD68^+^ cells, whereas CCR2^+^ macrophages were defined as CX3CR1-GFP^+^ CCR2-RFP^+^ CD68^+^ cells. Representative higher-magnification images with autofluorescence demonstrated localization of these macrophages within the myocardial interstitium rather than within vascular lumens. Exercise increased CCR2^+^ macrophages whereas CCR2^−^ macrophages decreased in the heart (LV and RV combined) (Figures 2B,C). When examining individual ventricular chambers, CCR2^+^ macrophages increased in the LV (Figure 2E) with a trend towards increase in the RV (Figure 2H). In both ventricles, the ratio of CCR2^+^ to CCR2^-^ macrophages significantly increased in VE mice compared to sedentary controls (Figures 2D,G,J).

When stratified by sex, the directionality of the CCR2^+^ to CCR2^-^ macrophage ratio was consistent across all groups. Notably, CCR2^-^ macrophages trended downward in all groups except the female LV (Supplementary Figure 3). Interestingly, the female LV was also the chamber in which we did not observe increases in LV mass or CSA, which raises the possibility that CCR2^-^ macrophages are protective against hypertrophy. These findings suggest that exercise alters the balance of CCR2^+^ and CCR2^−^ cardiac macrophages and implicates CCR2^+^ macrophages in exercise-associated cardiac hypertrophy. However, we did not perform lineage tracing or fate-mapping studies and therefore cannot determine whether the expanded CCR2^+^ macrophage population originated from circulating monocyte recruitment, local phenotypic transition, or expansion of pre-existing resident populations. Importantly, Ki67 immunostaining did not demonstrate increased proliferation of CCR2^+^ macrophages within either ventricle (Supplementary Figure 4), suggesting that local proliferation alone is unlikely to explain the observed increase.

### 3.3 CCR2^+^ macrophages are necessary for exercise-induced cardiac remodeling

We have demonstrated that prolonged (≥15 weeks) VE results in cardiac hypertrophy compared to sedentary conditions and increases CCR2^+^ to CCR2^-^ macrophage ratio in both LV and RV. Based on these findings, we hypothesized that VE-induced increases in cardiac CCR2^+^ macrophages are required for exercise-induced cardiac remodeling (Figure 2K). To assess this hypothesis, we employed CCR2^DTR/+^ mice in Cohort 3 and depleted CCR2^+^ macrophages throughout the course of exercise by administering diphtheria toxin (DT; Supplementary Figure 5). To demonstrate efficacy of our model, we performed spectral flow cytometry on CCR2^DTR/+^ and CCR2^+/+^ heart and identified a 70% reduction in cardiac CCR2^+^ macrophages with minimal disruption to other immune compartments (non-significant changes: NK Cells, T-cells, Eosinophils; significant decrease to B-cells) (Supplementary Figure 5).

**FIGURE 3:**
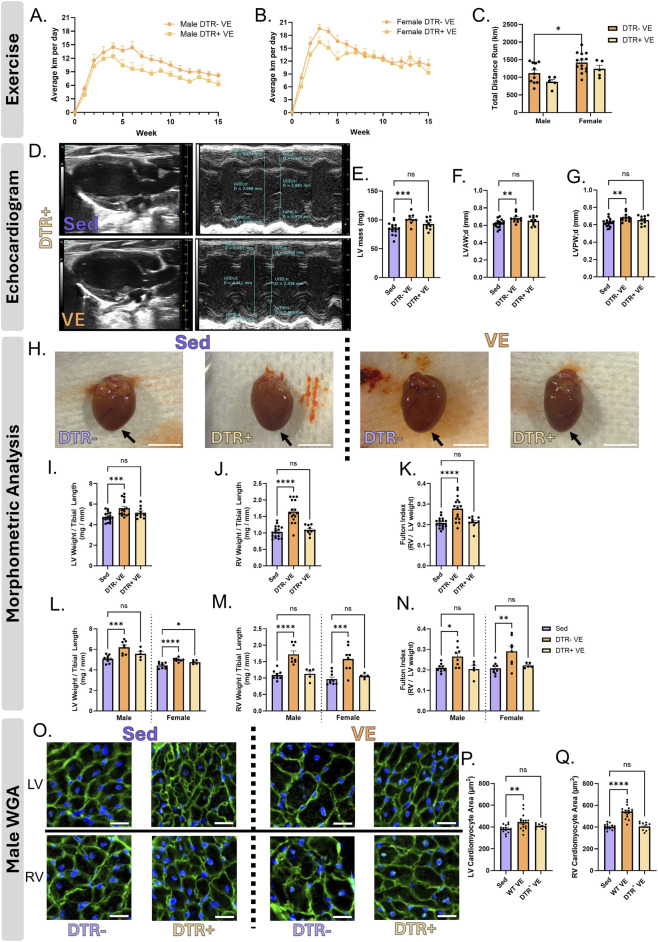
CCR2^+^ macrophages are required for voluntary exercise induced cardiac hypertrophy Average distance run per day for **(A)** male and **(B)** female VE DTR^−^ and DTR^+^ mice. Two-way repeated measures ANOVA with Sidak’s multiple comparison. **(C)** Total distance ran over 15 weeks (F effect of sex: p = 0.002, CCR2^+^ depletion: p = 0.037); n = 13–15/DTR^−^ group and 5/DTR^+^ group. **(D)** Representative images of LV echocardiogram parasternal long axis (left) and m-mode (right) of DTR^+^ female sedentary and VE. Echocardiogram measurements of **(E)** LV mass, **(F)** LVAW; d, and **(G)** LVPW; d of sedentary (combined DTR^−^ and DTR^+^), DTR^−^ VE, and DTR^+^ VE; n = 9–15/group. **(H)** Representative gross heart image of sedentary and VE groups. Arrow depicts differences in heart apex cardiac remodeling. Scale bar = 5 mm. Tibial length normalized **(I)** LV and **(J)** RV weights as well as **(K)** Fulton index of sedentary, DTR^−^ VE, and DTR^+^ VE. Male and female split of tibial length normalized weights of **(L)** LV and **(M)** RV weights as well as **(N**) Fulton index of sedentary, DTR^−^ VE, and DTR^+^ VE. **(O)** Representative LV and RV images of WGA (green) immunofluorescence of sedentary and VE groups. Scale bar = 20 µm. **(P)** LV and **(Q)** RV cardiomyocyte cross-sectional area of sedentary, DTR^-^ VE, and DTR^+^ VE; n = 10–18/group with equal splits of male and female mice. Data represented as mean ± SEM. **(C)** Two-way ANOVA with Sidak’s multiple comparison; **(E,F, I–N, P,Q)** One-way ANOVA with Dunnett’s multiple comparison. *p < 0.05, **p < 0.01, ***p < 0.001, ****p < 0.001. ns = nonsignificant. Diphtheria toxin receptor = DTR. Abbreviations as in Figures 1, 2.

To ensure that CCR2^+^ macrophage depletion did not alter the exercise stimulus, running behavior was assessed across all cohorts. We found no significant differences in running distance due to CCR2^+^ depletion in either sex (Figures 3A,B). Consistent with our prior findings, DTR^−^ females ran significantly farther than DTR^−^ males, whereas DTR^+^ females showed a trend toward greater running distance compared with DTR^+^ males (Figure 3C). Additionally, DTR^+^ mice maintained normal circadian running patterns during the 10-h active period, indicating that macrophage depletion did not impair voluntary exercise performance.

We next assessed whether CCR2^+^ macrophage depletion altered baseline (sedentary) cardiac morphology. With the exception of one observation where DTR^+^ female sedentary mice had lower LV mass compared with DTR^−^ sedentary females (Supplementary Figure 6H), we did not identify any differences in morphometric or echocardiographic measurements between sedentary DTR^+^ mice in Cohort 3 and sedentary DTR^−^ mice in previous cohorts (Supplementary Figure 6). Because baseline differences were minimal, DTR^+^ sedentary mice were combined with all other sedentary controls for subsequent analyses.

We then examined whether CCR2^+^ macrophage depletion would mitigate VE-induced cardiac hypertrophy (Figure 3). Similar to findings described earlier, echocardiography showed that DTR^−^ VE mice had increased LV mass, LVAW; d, and LVPW; d compared to sedentary DTR^−^ mice. In contrast, these parameters were not increased in DTR^+^ VE mice, which resembled sedentary controls (Figures 3D–G). No other echocardiographic measurements differed significantly between groups (Supplementary Figures 7, 8).

Representative gross heart morphology of DTR^−^ and DTR^+^ groups is shown in Figure 3H. Consistent with the echocardiographic findings, significant LV remodeling was only observed in DTR^−^ VE mice compared to sedentary controls, whereas DTR^+^ VE mice did not differ from sedentary mice (Figure 3I). CCR2^+^ macrophage depletion resulted in a significantly lower RV weight normalized to tibial length in DTR^−^ VE mice, with no difference compared to the sedentary group (Figure 3J). A similar pattern was observed with the Fulton index (Figure 3K). Together this demonstrates that despite maintaining the same level of exercise, CCR2^+^ macrophage depletion prevented RV hypertrophy and remodeling.

When stratified by sex, significant LV remodeling was only observed in male DTR^−^ VE mice and not DTR^+^ VE mice, whereas females demonstrated LV hypertrophy in both DTR^−^ and DTR^+^ VE groups (Figure 3L). This apparent female LV DTR^+^ VE hypertrophy could be explained by the previously noted reduction in LV hypertrophy in DTR^+^ female sedentary mice, as comparison of DTR^−^ sedentary and DTR^+^ VE females revealed no significant difference in LV hypertrophy (Supplementary Figure 6H). In both males and females, CCR2^+^ depletion prevented RV exercise-induced hypertrophy, reflected in normalized RV and Fulton index measures (Figures 3M,N).

We once again assessed whether the increase in heart weight was due to cardiomyocyte hypertrophy using WGA cardiomyocyte CSA analysis. Representative images of male DTR^−^ and DTR^+^ groups are shown in Figure 3O. In both LV and RV, only DTR^−^ VE mice had significantly increased cardiomyocyte CSA (Figures 3P,Q); thus, exercise-induced cardiomyocyte CSA increase was prevented with CCR2^+^ depletion. In sex-stratified analyses, females did not show differences in LV cardiomyocyte CSA between any groups (Supplementary Figure 7U). In contrast, CCR2^+^ depletion in males prevented the exercise-induced LV cardiomyocyte CSA increase. However, in both males and females, CCR2^+^ depletion prevented exercise-induced increase in RV cardiomyocyte CSA (Supplementary Figure 7V).

To assess whether prolonged exercise induces maladaptive remodeling within the heart, we quantified cardiac fibrosis and stiffness. We found no significant increase in fibrosis in the heart (LV + RV), LV, or RV (Supplementary Figures 9A–D). Using atomic force spectroscopy to measure cardiomyocyte stiffness (Supplementary Figure 9E), we did not observe an effect of VE on stiffness in either ventricle. Interestingly, we identified that CCR2^+^ depletion decreased LV cardiomyocyte stiffness in both the sedentary and VE groups and observed a trend of CCR2^+^ depletion decreasing RV cardiomyocyte stiffness in female sedentary but not VE mice (Supplementary Figures 9F–I). To further characterize the nature of exercise-induced remodeling, we performed quantitative PCR analysis of canonical hypertrophic markers. Expression of Myh6, Myh7, Nppa, and Nppb was assessed to distinguish physiologic from pathologic remodeling programs (Supplementary Figure 10). These data provide molecular context for the observed structural changes and suggest that CCR2^+^ macrophages are associated with activation of physiologic cardiomyocyte hypertrophic gene expression, although the precise signaling mechanisms remain to be defined. In summary, our findings demonstrate that VE induces cardiac hypertrophy in both sexes, with LV and RV hypertrophy in males and more significant RV hypertrophy in females. This hypertrophy correlated with an increased CCR2^+^ to CCR2^-^ macrophage ratio. Depletion of CCR2^+^ macrophages prevented LV and RV hypertrophy in both sexes. Lastly, although hypertrophy and increased mass were consistently observed, no fibrosis or increased cardiomyocyte stiffness were detected.

## 4 Discussion

Exercise is widely recognized as a cornerstone of cardiovascular health, with substantial evidence demonstrating its capacity to improve cardiac function, enhance vascular compliance, and reduce long-term cardiovascular risk. As a result, an “exercise prescription” is frequently recommended for patients with a variety of cardiopulmonary disorders. However, accumulating data also indicates that excessive or high-intensity endurance exercise is associated with maladaptive consequences, including cardiac fibrosis, arrhythmias, and accelerated coronary artery disease (Franklin et al., 2020). These observations support the concept of a dose-response relationship in which exercise confers cardiovascular benefit up to a threshold, beyond which structural and electrophysiological abnormalities may develop.

In this study, we used voluntary exercise (VE) in mice as a model to examine the cardiac remodeling process induced by sustained, physiologic physical activity. Our goals were twofold: (1) to characterize the immune response to prolonged exercise, and (2) to assess the hypothesis that CCR2^+^ macrophages play a role in mediating exercise-induced cardiac remodeling.

Our findings demonstrate that VE reliably induced cardiac remodeling consistent with previously well-described adaptive hypertrophic response to endurance exercise (Lerchenmuller et al., 2022; Allen et al., 2001; Eder et al., 2022; Liu et al., 2015; Hastings et al., 2022). This hypertrophic remodeling is typically regarded as a physiologic adaptation that supports enhanced cardiac output during sustained physical activity. Consistent with this interpretation, we did not observe evidence of myocardial fibrosis in either ventricle in VE mice, supporting that the structural changes represent physiologic adaptation rather than pathologic remodeling.

Several findings support the interpretation that the observed remodeling is predominantly physiologic, including preserved systolic function and the absence of increased fibrosis. In addition, molecular profiling of hypertrophic markers (Myh6, Myh7, Nppa, and Nppb) provides further context for distinguishing adaptive versus maladaptive remodeling. However, we did not assess functional reserve (e.g., exercise capacity under stress or VO_2_ max) and therefore cannot exclude more subtle impairments in cardiac performance following CCR2^+^ macrophage depletion.

Notably, we observed that VE-induced cardiac hypertrophy was accompanied by a shift in the myocardial immune landscape, with a significant increase in the ratio of CCR2^+^ to CCR2^-^ macrophages in both ventricles compared with sedentary controls. While CCR2^+^ macrophages have previously been well-characterized as pro-inflammatory cells that can promote maladaptive remodeling in disease contexts, our findings suggest a more nuanced role in the context of physiologic exercise. In our VE model, remodeling occurred without fibrosis or increased cardiomyocyte stiffness, consistent with a physiologic response; thus, CCR2^+^ depletion-mediated prevention of hypertrophy may represent blunting of an adaptive program rather than protection from pathology. However, because CCR2^+^ macrophages can drive inflammatory remodeling in disease models, it remains possible that their contribution to exercise remodeling reflects a shared pathway that could become maladaptive with higher or excessive exercise “dose,” aging, or cardiometabolic stress.

While our data demonstrates that CCR2^+^ macrophages are required for exercise-induced cardiac hypertrophy, the mechanisms by which these cells influence cardiomyocyte growth remain undefined. One possibility is that CCR2^+^ macrophages promote hypertrophy through paracrine signaling, such as secretion of growth factors (e.g., IGF-1) or cytokines that modulate cardiomyocyte gene expression. Alternatively, macrophage-cardiomyocyte interactions may occur through direct cell-cell contact or modulation of the extracellular microenvironment. Future studies using cell-specific transcriptomic or proteomic approaches will be required to delineate these pathways. Additionally, exercise may result in upstream changes such as alterations in capillary networks (Molmen et al., 2025; De Ciuceis et al., 2023) not assessed within this study.

Additionally, we observed that mitigating exercise-induced hypertrophy through depletion of CCR2^+^ macrophages did not reduce the total voluntary running distance within sex. Although we did not specifically assess maximal running capacity, these findings suggest that the presence of CCR2^+^ macrophages is not required for mice to engage in or maintain typical voluntary exercise behavior. Defining the net impact of CCR2^+^ macrophage depletion will require studies that quantify functional reserve (e.g., treadmill VO_2_max/capacity, cardiac output during stress) and myocardial energetics.

Of particular interest, we found that CCR2^+^ macrophage depletion prevented exercise-associated increase in spleen mass, mirroring our cardiac remodeling findings. The spleen has been proposed to provide functional benefit during hypoxia by mobilizing erythrocytes (Lodin-Sundstrom et al., 2021; Stewart et al., 2003; Laub et al., 1993), with high-altitude populations (Brutsaert et al., 2024; Schagatay et al., 2020) and endurance athletes (Holmstrom et al., 2021) exhibiting larger resting spleen volumes. Given that this study was conducted at ∼5,280 ft (our center’s elevation), splenic adaptations may not be solely immune-specific but could also reflect enhanced erythrocyte storage and mobilization to support increased oxygen demand. Consistent with this concept, some evidence suggests that heart failure patients with greater spleen volumes demonstrated better exercise tolerance (Hiraiwa et al., 2022b), although this increased volume may reflect expanded monocytes populations implicated in heart failure pathogenesis (Hiraiwa et al., 2022a; Prabhu, 2018; Fujinami et al., 2018; Swirski et al., 2009). It remains unclear whether our observations reflect changes in splenic erythrocyte and/or immune cell composition; however, the cardio-splenic axis remains an intriguing area for future investigation.

An important unresolved question is the origin of the expanded CCR2^+^ macrophage population observed with exercise. CCR2^+^ macrophages are generally thought to arise from circulating monocytes; however, we were only able to demonstrate that local proliferation of CCR2^+^ macrophages was not increased. Future studies using lineage tracing or other fate mapping techniques will be required to clarify the relative contributions of these mechanisms. An additional limitation is that we did not characterize circulating peripheral blood monocyte subsets before or after exercise. Prior studies have demonstrated exercise-associated alterations in CCR2 expression across circulating classical, intermediate, and non-classical monocyte populations (Blanks et al., 2020; Blanks et al., 2022; Kawanishi et al., 2010). As a result, we cannot determine whether the increased cardiac CCR2^+^ macrophages observed in this study reflect altered monocyte recruitment dynamics, changes in circulating CCR2 expression, or local myocardial immune adaptation. Future studies integrating peripheral blood immunophenotyping with cardiac lineage tracing approaches will be important to resolve these relationships.

An unexpected but particularly compelling observation was that depletion of CCR2^+^ macrophages reduced cardiomyocyte stiffness in sedentary mice, with a more noticeable trend in females compared to males. This sex-dependent difference is notable considering emerging research linking macrophage-driven inflammation and fibrosis to the pathophysiology of heart failure with preserved ejection fraction (HFpEF), a condition disproportionately affecting women. Prior work has shown that CCR2^+^ macrophages contribute to myocardial inflammation and fibrosis in HFpEF models, and that their depletion can improve both ventricular compliance and hemodynamic performance (Raman et al., 2025). In light of increasing recognition of interactions between biologic sex, sex hormones, and immune function in the heart (Gu et al., 2025), these findings raise the possibility that sex-dependent immune signaling pathways may influence myocardial regulation and ventricular mechanics. Although investigation of HFpEF mechanisms was beyond the scope of our study, our findings add to a growing body of evidence implicating CCR2^+^ macrophages in sex-specific cardiac responses and suggest avenues for future investigation.

Despite these insights, our study has several important limitations. First, we were unable to control or quantify physical activity occurring outside the running wheel. It is therefore possible that baseline cage activity contributed to variability in exercise exposure. Second, VE required single housing, which is known to induce varying degrees of psychosocial stress in mice and may therefore influence metabolic, hormonal, or immune outcomes in ways that differ from group-housed animals. Third, sex-specific differences in cardiac remodeling were observed, particularly with respect to RV hypertrophy in females. However, this study was not powered to definitively assess sex-dependent mechanisms, and these findings should be interpreted cautiously. The absence of fibrosis or increased cardiomyocyte stiffness suggests that overt pathological remodeling is unlikely, although differences in cardiopulmonary loading conditions or immune responses may contribute and warrant further investigation. Single housing, space limitations, and the prolonged duration of the experimental protocol collectively restricted the total number of animals that could be included. As a result, some subtle or sex-dependent effects may not have reached statistical significance. Although we used CCR2^DTR/+^ mice to deplete CCR2-expressing cells, CCR2 is not exclusive to a single macrophage subset and may also be expressed by other myeloid populations. Additionally, although immunofluorescence demonstrated co-localization of CCR2 reporter expression with the macrophage marker CD68 and interstitial myocardial localization, CCR2 expression is not entirely specific to macrophages and may also occur in other myeloid populations under certain conditions. Furthermore, depletion of CCR2^+^ could result in more dramatic immune shifts than previously realized. Consistent with this, our spectral flow cytometry analysis following diphtheria toxin administration confirms depletion of CCR2^+^ cells without shifts in NK or T-cells but did demonstrate reduction in B-cells. Thus, we cannot fully attribute the observed phenotype solely to a single macrophage subset, highlighting the need for more refined, lineage-specific approaches.

## 5 Conclusion

Overall, our findings demonstrate that CCR2^+^ cardiac macrophages contribute importantly to exercise-induced cardiac remodeling and highlight a previously underappreciated role for immune-cardiac interactions in physiologic adaptation to exercise. To our knowledge, relatively few studies have systematically examined LV and RV remodeling in parallel during chronic voluntary exercise while also incorporating both sexes in sufficient numbers to detect sex-specific differences. Our findings underscore the complex interplay between the immune system and myocardial adaptation to physiologic stress, and they highlight areas where further mechanistic and sex-focused studies are warranted.

## Data Availability

The raw data supporting the conclusions of this article will be made available by the authors, without undue reservation.
