# Supplementary material for: CCR2+ macrophages are required for exercise-induced cardiac remodeling
Source: Front Pharmacol. 2026 Jun 25;17:1844427. doi: 10.3389/fphar.2026.1844427 (PMC13345881; doi:10.3389/fphar.2026.1844427)

Supplementary Figure 1. Additional running and morphometrics, echocardiography, and LV cardiomyocyte cross-sectional analysis. (A) Actogram depicting individual running for one day of Cohorts 1,2, and 3 mice during week 4. Hours 8–18 (blue) represents lights-off, active period. **(B)** Body weights of male and female sedentary and VE mice at pre or post intervention. Two-way repeated measures ANOVA with Sidak's multiple; Male n = 8-14 / group. Female n = 7-15 / group. Unnormalized (**C**) spleen weight, (**D**) tibial normalized spleen weight, and (**E**) tibial normalized spleen weight split by sex of sedentary and VE groups; n = 12-16 / group with equal splits of male and female mice. Echocardiogram measurements of (**F**) heart rate, (**G**) LV systolic anterior wall thickness (LVAW;s), (**H**) LV systolic posterior wall thickness (LVPW;s), (**I and J**) LV internal diameter in diastole and systole (LVID;d and LVID;s, respectively), (**K**) mitral annulus active wave peak velocity (A'), (**L**) mitral annulus early wave peak velocity (E'), (**M**) mitral valve active wave peak velocity (A), (**N**) mitral valve early wave peak velocity (E), (**O**) ejection fraction percent (EF%), (**P and Q**) LV volume in diastole and systole (LV Vol;d and LV Vol;s, respectively), (**R**) E'/A', (**S**) E/A, and (**T**) E/E'; n = 11 per group. **(U)** Representative LV images of WGA (green) immunofluorescence of sedentary and VE split by sex. Scale bar = 20 μm. LV cardiomyocyte cross-sectional area of (**V**) combined and (**W**) split male and female; n = 12-16 / group with equal splits of male and female mice. Data represented as mean ± SEM. Unpaired t-test used unless designed otherwise. **(C and D, F to K, M to Q, S and T,V)** Unpaired Student's t-test; **(L,R)** Mann-Whitney U test; (**E,W**) Two-way ANOVA with Sidak's multiple comparison. $^{**}p < 0.01$, $^{***}p < 0.001$, $^{****}p < 0.001$. Abbreviations as in Figure 1.

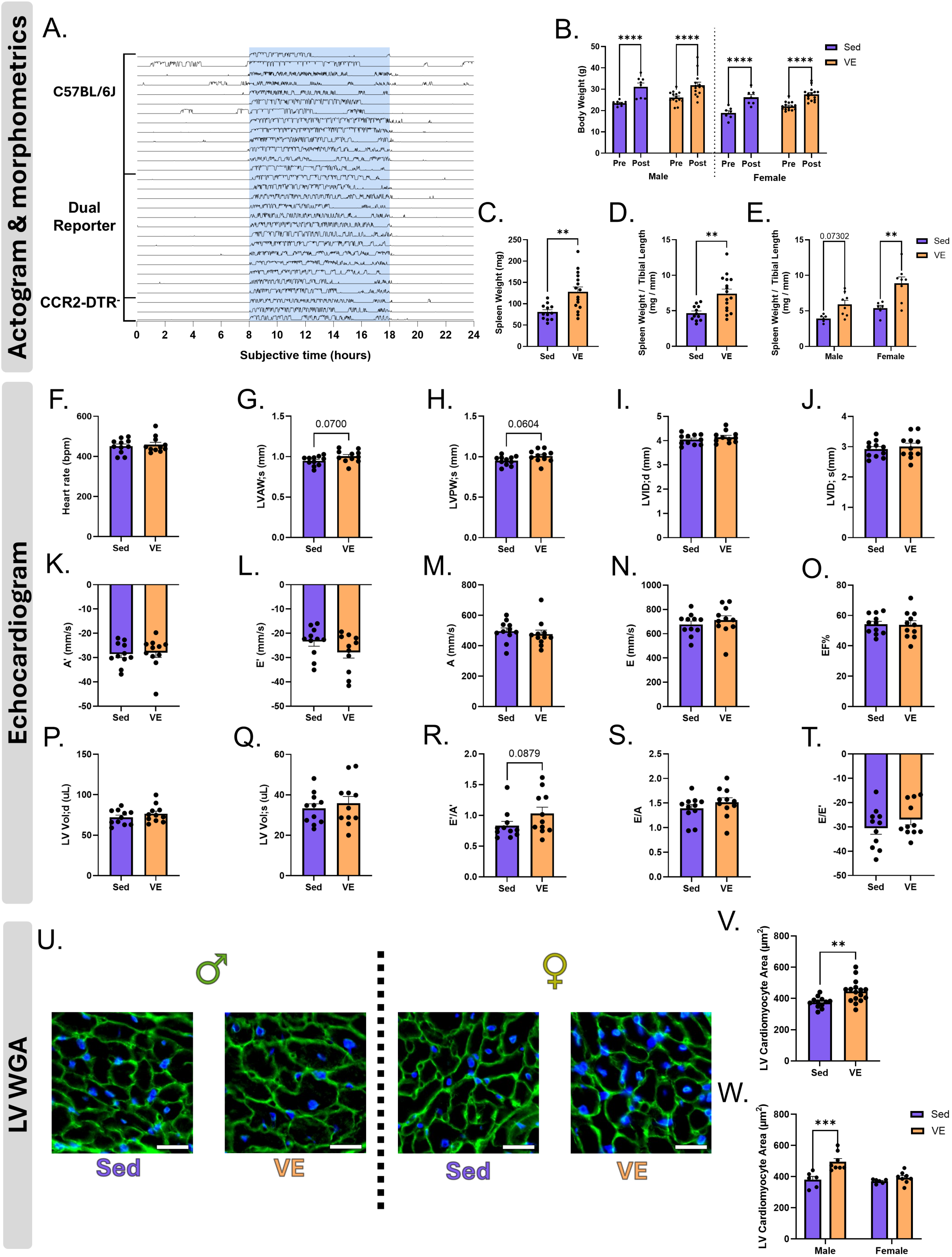

Actogram & morphometrics
A.
C57BL/6J
Dual Reporter
CCR2-DTR-
Subjective time (hours)
B.
Body Weight (g)
Sed
VE
Pre Post
Male
Female
C.
Spleen Weight (mg)
D.
Spleen Weight / Tibial Length (mg / mm)
E.
0.07302
Echocardiogram
F. Heart rate (bpm)
G. LVAW;s (mm) 0.0700
H. LVPW;s (mm) 0.0604
I. LVID;d (mm)
J. LVID; s(mm)
K. A' (mm/s)
L. E' (mm/s)
M. A (mm/s)
N. E (mm/s)
O. EF%
P. LV Vol;d (uL)
Q. LV Vol;s (uL)
R. E'/A' 0.0879
S. E/A
T. E/E'
LV WGA
U.
V.
LV Cardiomyocyte Area (μm²)
W.

Supplementary Figure 2. Echocardiography of male versus female. Echocardiogram of sedentary (sed) and VE split by male and female. Measurements shown are **(A)** LVAW;d, **(B)** LVAW;s, **(C)** LVPW;d, **(D)** LVPW;s, **(E)** heart rate, **(F)** LV mass, **(G)** LVID;d (F effect of sex: p = 0.016)**, (H)** LVID;s, **(I)** A' ,**(J)** E',**(K)** LV Vol;d, (F effect of sex: p = 0.016), **(L)** LV Vol;s, **(M)** EF%, **(N)** E'/A', **(O)** E/A, and **(P)** E/E'; n = 5–6 / group. Data represented as mean ± SEM. (**A to P**) Two-way ANOVA with Sidak's multiple comparison. *p < 0.05, **p < 0.01. $ p < 0.05 male vs female F effect. Abbreviations as in Figure 1 and Supplementary Figure 1.

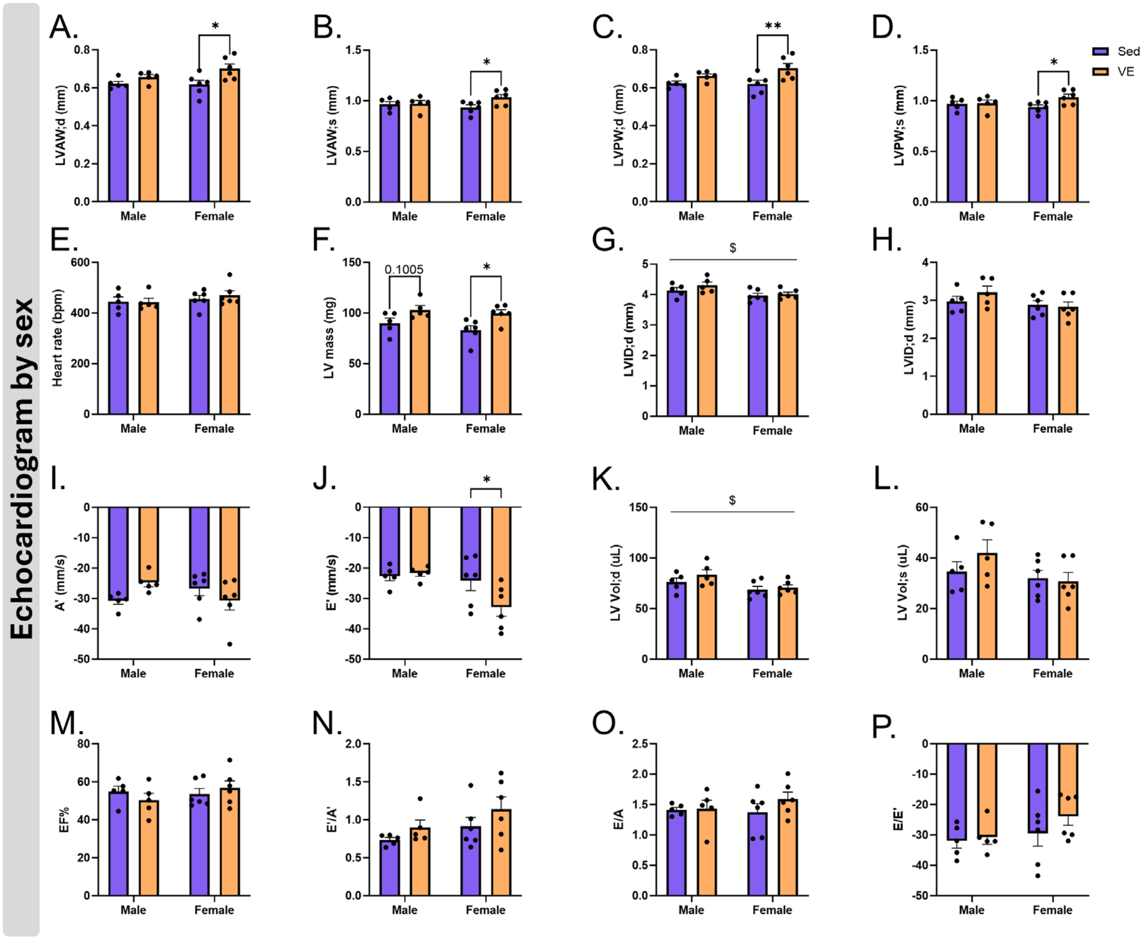

Supplementary Figure 3. Sex-stratified cardiac CCR2$^+$ and CCR2$^-$ macrophage composition. Quantification of sedentary and VE heart (LV and RV combined) CCR2$^+$ macrophages in **(A)** males and **(J)** females, CCR2$^-$ macrophages in **(B)** male and **(H)** females, and ratio of CCR2$^+$ to CCR2$^-$ macrophages in **(C)** male and **(L)** females. Quantification of LV CCR2$^+$ macrophages in **(D)** males and **(M)** females, CCR2$^-$ macrophages in **(E)** males and **(N)** females, and ratio of CCR2$^+$ to CCR2$^-$ macrophages in **(F)** males and **(O)** females. Quantification of RV CCR2$^+$ macrophages in **(G)** males and **(P)** females, CCR2$^-$ macrophages in **(H)** males and **(Q)** females, and ratio of CCR2$^+$ to CCR2$^-$ macrophages in **(I)** males and **(R)** females; Male n = 5-6 per group. Female n = 4-7 / group. Data represented as mean ± SEM. (**A to K, M and N, P to R)** Unpaired Student's t-test; (**L and O)** Mann-Whitney U test. $*p < 0.05$, $**p < 0.01$. Abbreviations as in Figure 1 and Figure 2.

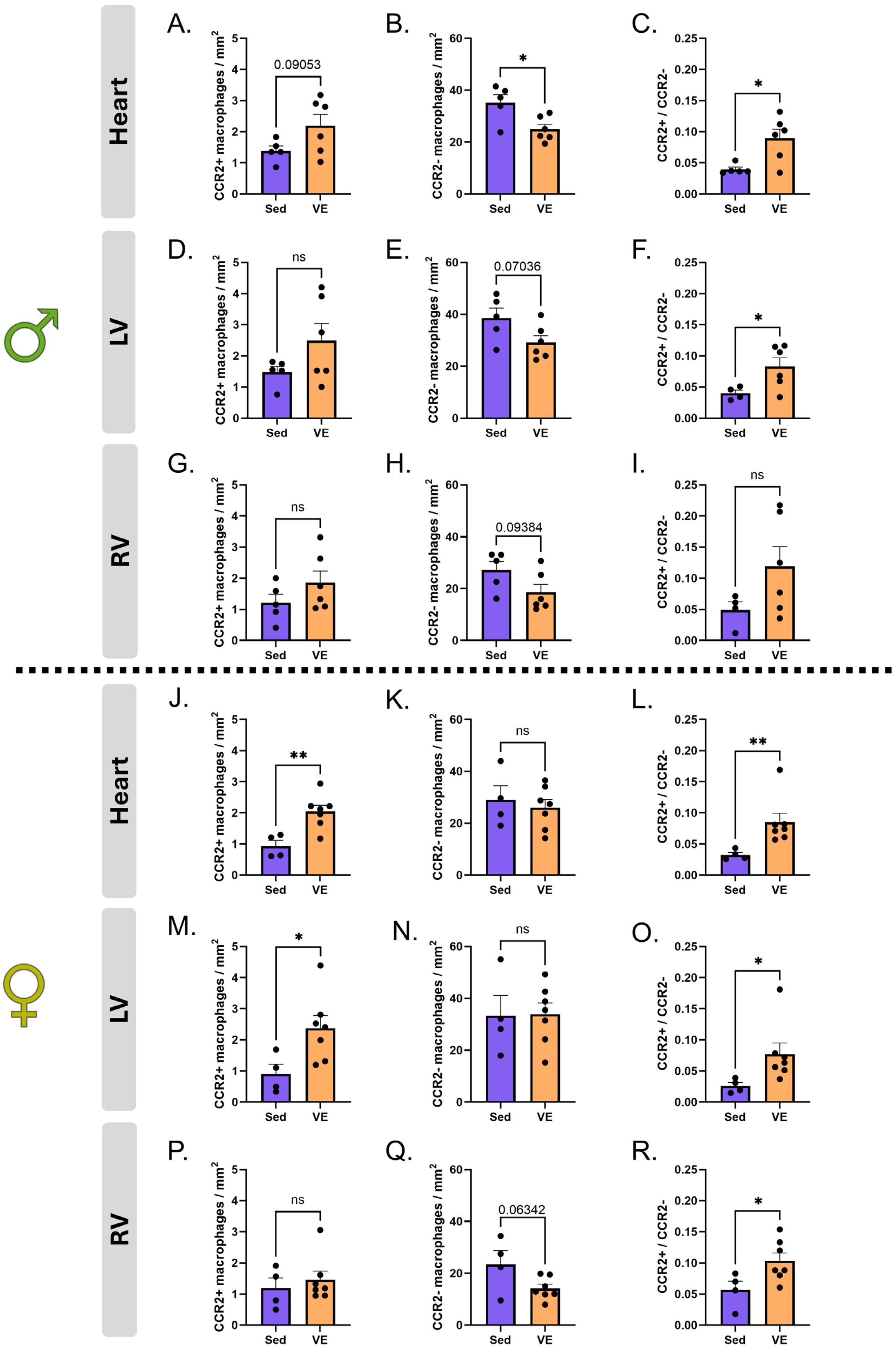
Heart
LV
RV
Heart
LV
RV
A.
B.
C.
D.
E.
F.
G.
H.
I.
J.
K.
L.
M.
N.
O.
P.
Q.
R.
CCR2+ macrophages / mm2
CCR2- macrophages / mm2
CCR2+ / CCR2-
0.09053
0.07036
0.09384
0.06342
ns
*
**
Sed
VE

Supplementary Figure 4. Voluntary exercise did not promote cardiac CCR2$^+$ macrophage proliferation. (A) Representative images of immunofluorescence of Cohort 2 (DR mice) with DAPI (blue), CX3CR1 - GFP (green), CCR2 - RFP (red), and CD68 (Ki67) of sedentary and VE. Scale bar = 50 µm. Quantification of percent (**B**) heart (LV and RV combined), (**C**) LV, and (**C**) RV CCR2+ Ki67+ macrophages. Male n = 1-3 and female n = 3-4 / group. Data represented as mean ± SEM. (**B and C)** Unpaired Student's t-test; (**D)** Mann-Whitney U test. Abbreviations as in Figure 1 and Figure 2.

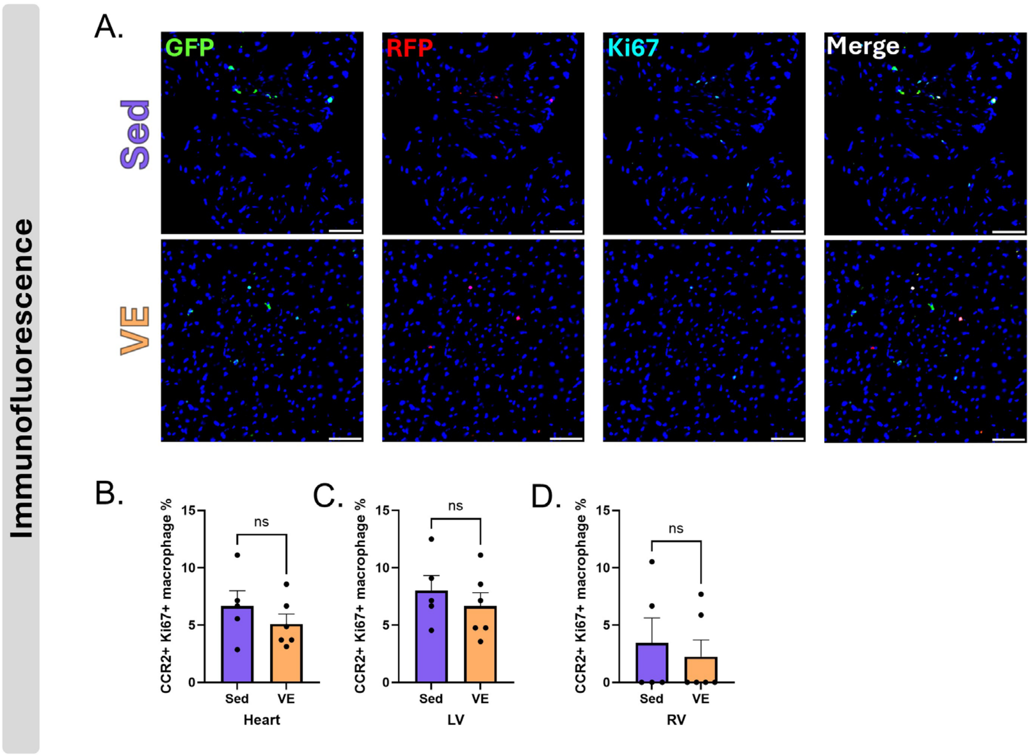

Supplementary Figure 5. Spectral flow cytometry of DT mediated CCR2$^+$ depletion in cardiac immune profile. A separate cohort of sedentary female CCR2-DTR$^-$ (CCR2$^{+/+}$) and CCR2-DTR$^+$ (CCR2$^{DTR/+}$) mice were given 6 total DT intraperitoneal injections over the course of two weeks with spectral flow cytometry occurring the day after the last injection. **(A)** Representative spectral flow gating for cardiac macrophages (Mac), CCR2$^+$ and CCR2$^-$ macrophages, and T and B cells. **(B)** Percent of live immune cells per single cell. Percent of **(C)** B cells, **(D)** T cells, **(E)** natural killer (NK) cells, **(F)** eosinophils, **(G)** macrophages, **(H)** CCR2$^+$ macrophages, and **(I)** CCR2$^-$ macrophages per live immune cells. Female n = 3 / group. Data represented as mean ± SEM. (**B to I)** Unpaired Student's t-test; *p < 0.05, ***p < 0.001. Diphtheria toxin = DT. Abbreviations as in Figure 2 and 3.

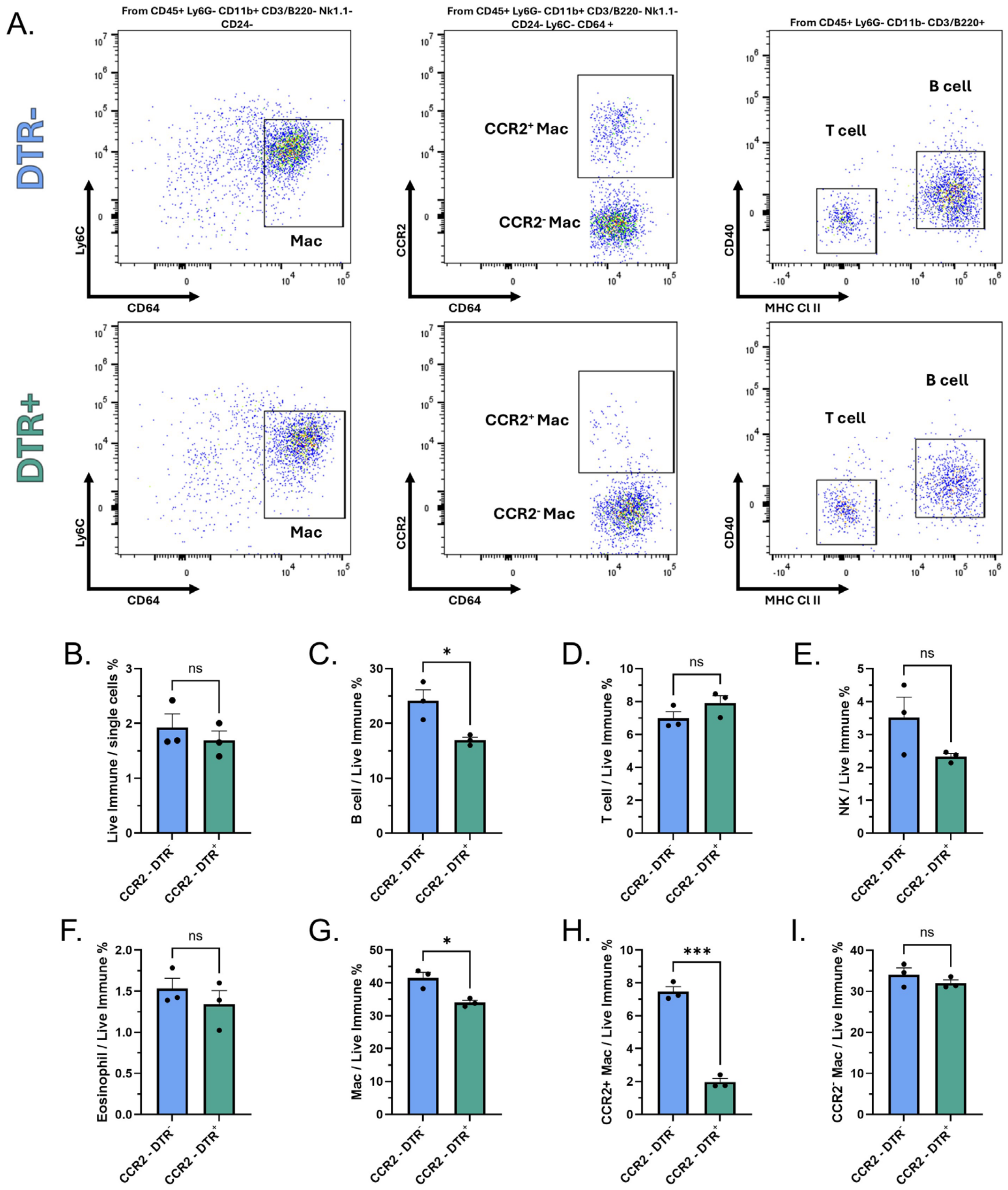
A.
DTR-
DTR+
From CD45+ Ly6G- CD11b+ CD3/B220- Nk1.1- CD24-
From CD45+ Ly6G- CD11b+ CD3/B220- Nk1.1- CD24- Ly6C- CD64 +
From CD45+ Ly6G- CD11b- CD3/B220+
Mac
CCR2+ Mac
CCR2- Mac
T cell
B cell
Ly6C
CD64
CCR2
CD40
MHC Cl II
B.
C.
D.
E.
F.
G.
H.
I.
Live Immune / single cells %
B cell / Live Immune %
T cell / Live Immune %
NK / Live Immune %
Eosinophil / Live Immune %
Mac / Live Immune %
CCR2+ Mac / Live Immune %
CCR2- Mac / Live Immune %
CCR2 - DTR-
CCR2 - DTR+
ns
*
***

Supplementary Figure 6. DTR$^-$ and DTR$^+$ comparison of echocardiography, morphometrics, and cardiomyocyte cross-sectional analysis. Echocardiogram measurements of (**A**) LV mass, (**B**) LVAW;d, and (**C**) LVPW;d of DTR$^-$ and DTR$^+$ sedentary and VE mice; n = 11 / DTR- group. n= 6–10 / DTR$^+$ group. Tibial length normalized (**D**) heart, (**E**) LV, and (**F**) RV weights as well as **(G)** Fulton index of DTR$^-$ and DTR$^+$ sedentary and VE mice. Male and female split of tibial length normalized weights of (**H**) LV and (**I**) RV weights as well as (**J**) Fulton index of DTR$^-$ and DTR$^+$ sedentary and VE mice. (**K**) LV and (**J**) RV cardiomyocyte cross-sectional area; n = 12–16 per DTR$^-$ group with equal splits of male and female mice. n = 6–10 / DTR$^+$ group with equal splits of male and female mice. Data represented as mean ± SEM. (**A to J**) Two-way ANOVA with Sidak's multiple comparison. *$p < 0.05$, **$p < 0.01$, ***$p < 0.001$, ****$p < 0.001$. ns = nonsignificant. Abbreviations as in Figure 1, Figure 2, and Figure 3.

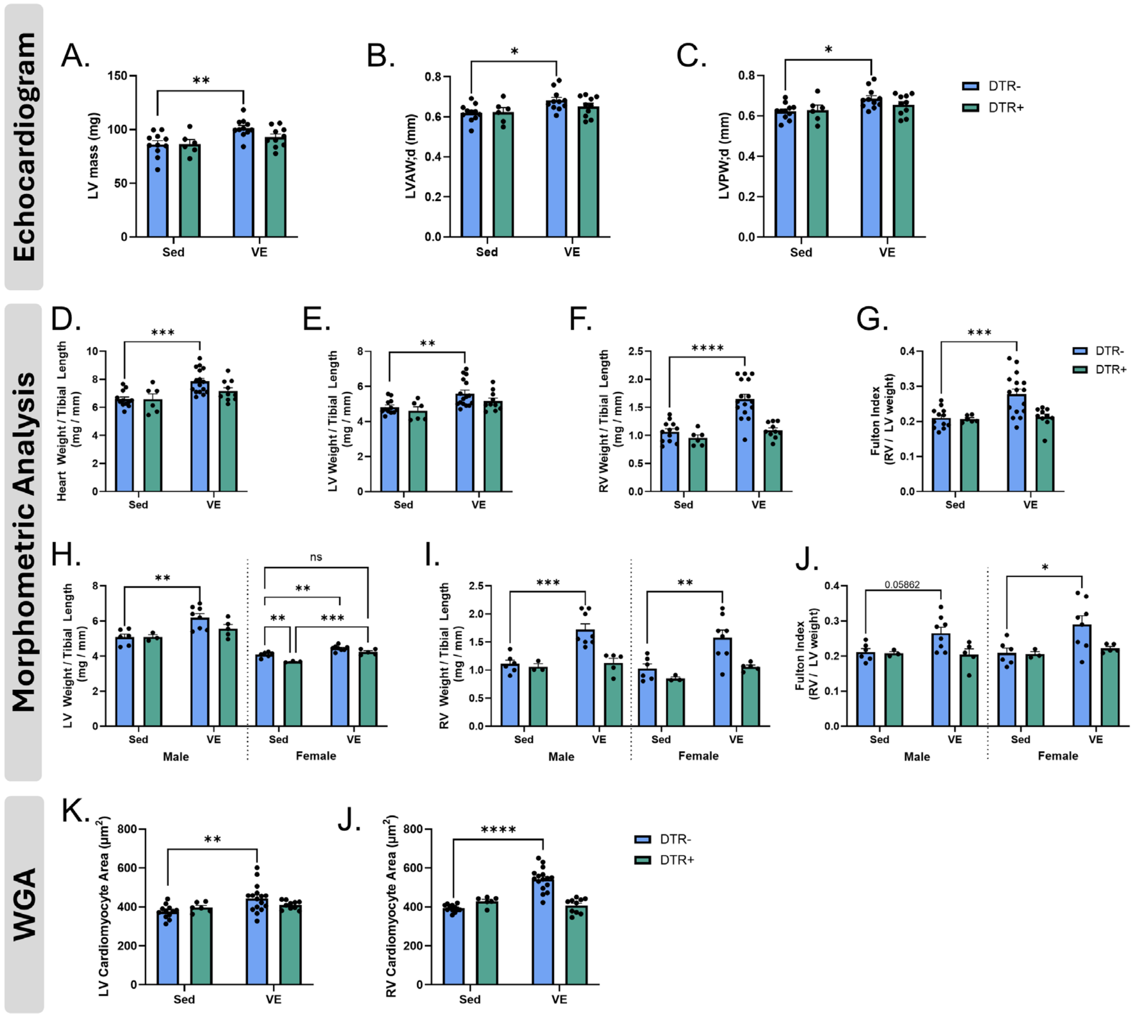

Supplementary Figure 7. Additional running and morphometrics, echocardiography, and cardiomyocyte cross-sectional analysis with DTR$^+$ groups. (A) Actogram depicting individual running for one day of all DTR$^-$ and DTR$^+$ mice during week 4. Hours 8-18 (blue) represents lights-off, active period. **(B)** Weights of male and female sedentary and VE DTR$^-$ and DTR$^+$ mice pre or post intervention. Three-way repeated measures ANOVA with Sidak's multiple comparison; Male n = 8-14 / DTR$^-$ group and n = 3-5 / DTR$^+$ group. Female n = 7-15 / DTR$^-$ group and n = 3-5 / DTR$^+$ group. **(C)** Spleen weight, **(D)** tibial normalized spleen weight, and **(E)** tibial normalized spleen weight split by sex of male and female sedentary and VE DTR$^-$ and DTR$^+$ mice; n = 12-16 per DTR$^-$ group and n = 6-10 / DTR$^+$ group with equal split of male and females. Echocardiogram measurements of **(F)** heart rate, **(G)** LVAW;s, **(H)** LVPW;s, **(I and J)** LVID;d and LVID;s, respectively, (**K**) A', (**L**) E', (**M**) A, (**N**) E, (**O**) EF%, (**P and Q**) LV Vol;d and LV Vol;s, respectively, (**R**) E'/A', (**S**) E/A, and **(T)** E/E' of sedentary and VE DTR$^-$ and DTR$^+$ mice; n = 11 / DTR$^-$ group and n = 6-10 / DTR$^+$ group. WGA (green) immunofluorescence **(U)** LV and **(V)** RV cardiomyocyte cross-sectional area analysis of split male and female sedentary and VE DTR$^-$ and DTR$^+$ mice; n = 6-8 / DTR$^-$ group and n = 3-5 / DTR$^+$ group. Data represented as mean ± SEM. (**C to V**) Two-way ANOVA with Sidak's multiple comparison. $^{**}p < 0.01$, $^{***}p < 0.001$, $^{****}p < 0.001$. Abbreviations as in Figure 1, Figure 3, and Supplementary Figure 1.

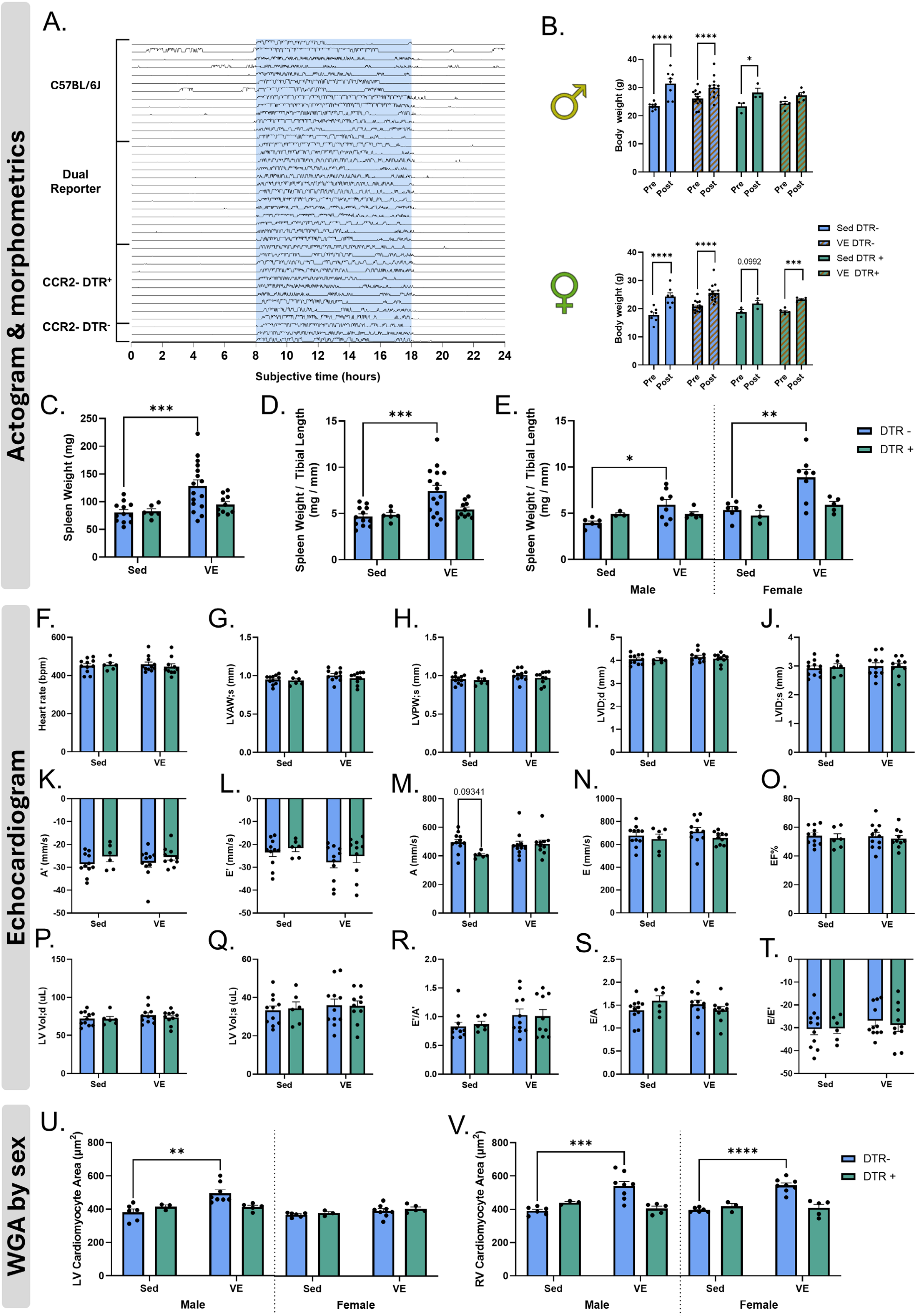
Actogram & morphometrics
A.
C57BL/6J
Dual Reporter
CCR2- DTR+
CCR2- DTR-
Subjective time (hours)
B.
Body weight (g)
Pre
Post
Sed DTR-
VE DTR-
Sed DTR +
VE DTR+
0.0992
C.
Spleen Weight (mg)
D.
Spleen Weight / Tibial Length (mg / mm)
E.
DTR -
DTR +
Sed
VE
Male
Female
Echocardiogram
F.
Heart rate (bpm)
G.
LVAW;s (mm)
H.
LVPW;s (mm)
I.
LVID;d (mm)
J.
LVID;s (mm)
K.
A' (mm/s)
L.
E' (mm/s)
M.
0.09341
A (mm/s)
N.
E (mm/s)
O.
EF%
P.
LV Vol;d (uL)
Q.
LV Vol;s (uL)
R.
E'/A'
S.
E/A
T.
E/E'
WGA by sex
U.
LV Cardiomyocyte Area (µm²)
V.
RV Cardiomyocyte Area (µm²)
DTR-
DTR +

Supplementary Figure 8. Sex-stratified echocardiography with DTR^+ groups. Echocardiogram of sedentary and VE DTR^- and DTR^+ split by male and female. Measurements shown are **(A)** LVAW;d, **(B)** LVAW;s, **(C)** LVPW;d, **(D)** LVPW;s, **(E)** A', **(F)** E', **(G)** A, **(H)** E (male F effect of $CCR2^+$ depletion: p = 0.0465), **(I)** EF%, **(J)** E'/A', **(K)** E/A, and **(L)** E/E'; n = 5–6 / DTR^- group. n = 3-5 / DTR^+ group. Data represented as mean ± SEM. **(A to L)** Two-way ANOVA with Sidak's multiple comparison. *p < 0.05, **p < 0.01, ***p < 0.001, ****p < 0.001. # p < 0.05 $CCR2^+$ depleted vs non-depleted F effect. Abbreviations as in Figure 1, Figure 3, and Supplementary Figure 1.

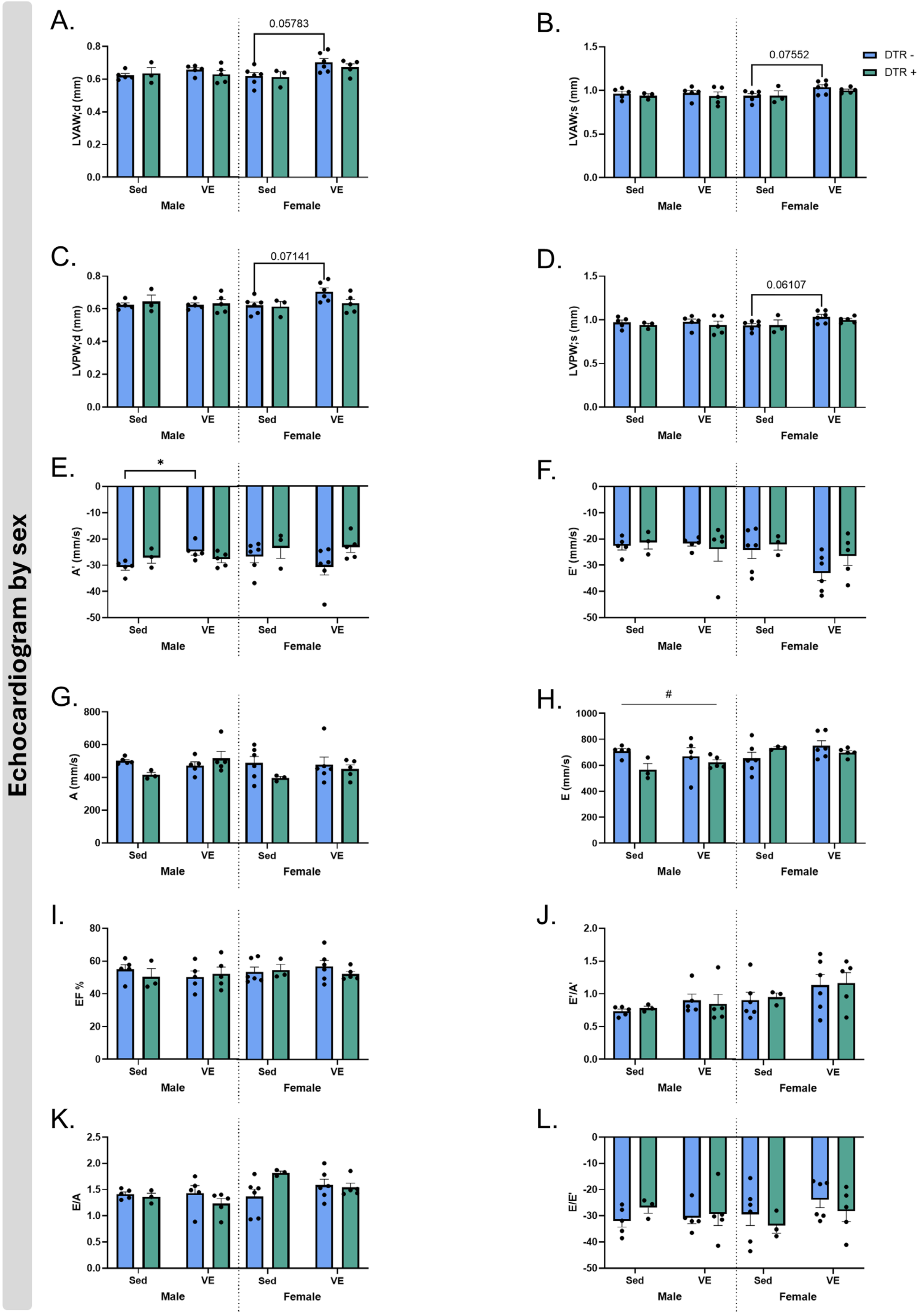
Echocardiogram by sex
A.
0.05783
LVAW;d (mm)
B.
0.07552
LVAW;s (mm)
DTR -
DTR +
C.
0.07141
LVPW;d (mm)
D.
0.06107
LVPW;s (mm)
E.
*
A' (mm/s)
F.
E' (mm/s)
G.
A (mm/s)
H.
#
E (mm/s)
I.
EF %
J.
E'/A'
K.
E/A
L.
E/E'
Sed
VE
Male
Female

Supplementary Figure 9. Tissue fibrosis and cardiomyocyte stiffness did not change with voluntary exercise. (A) Representative female RV fibrosis images of sedentary and VE DTR^- and DTR^+. Cohort 3 Pico Sirius Red (PSR) fibrosis percent of **(B)** heart (LV + RV), **(C)** LV, and **(D)** RV; n = 3-10 / group. **(E)** Representative AFM image with AFM probe tip contacting cardiomyocyte rich regions highlighted by cardiomyocyte nuclei (black arrow). Aggregated Cohort 2 and 3 Atomic Force Microscopy (AFM) of cardiomyocyte stiffness (Young's Modulus) of **(F-G)** LV (F effect of $CCR2^+$ depletion: $p = 0.026$) and **(H-I)** RV sedentary and VE DTR^- and DTR^+; n = 6-9 / group. Male and female split AFM of **(G)** LV and **(I)** RV sedentary and VE DTR^- and DTR^+. n = 2-6 / group. Data represented as mean ± SEM. **(B to I)** Two-way ANOVA with Sidak's multiple comparison. # $p < 0.05$ $CCR2^+$ depleted vs non-depleted F effect. Abbreviations as in Figure 1 and Figure 3.

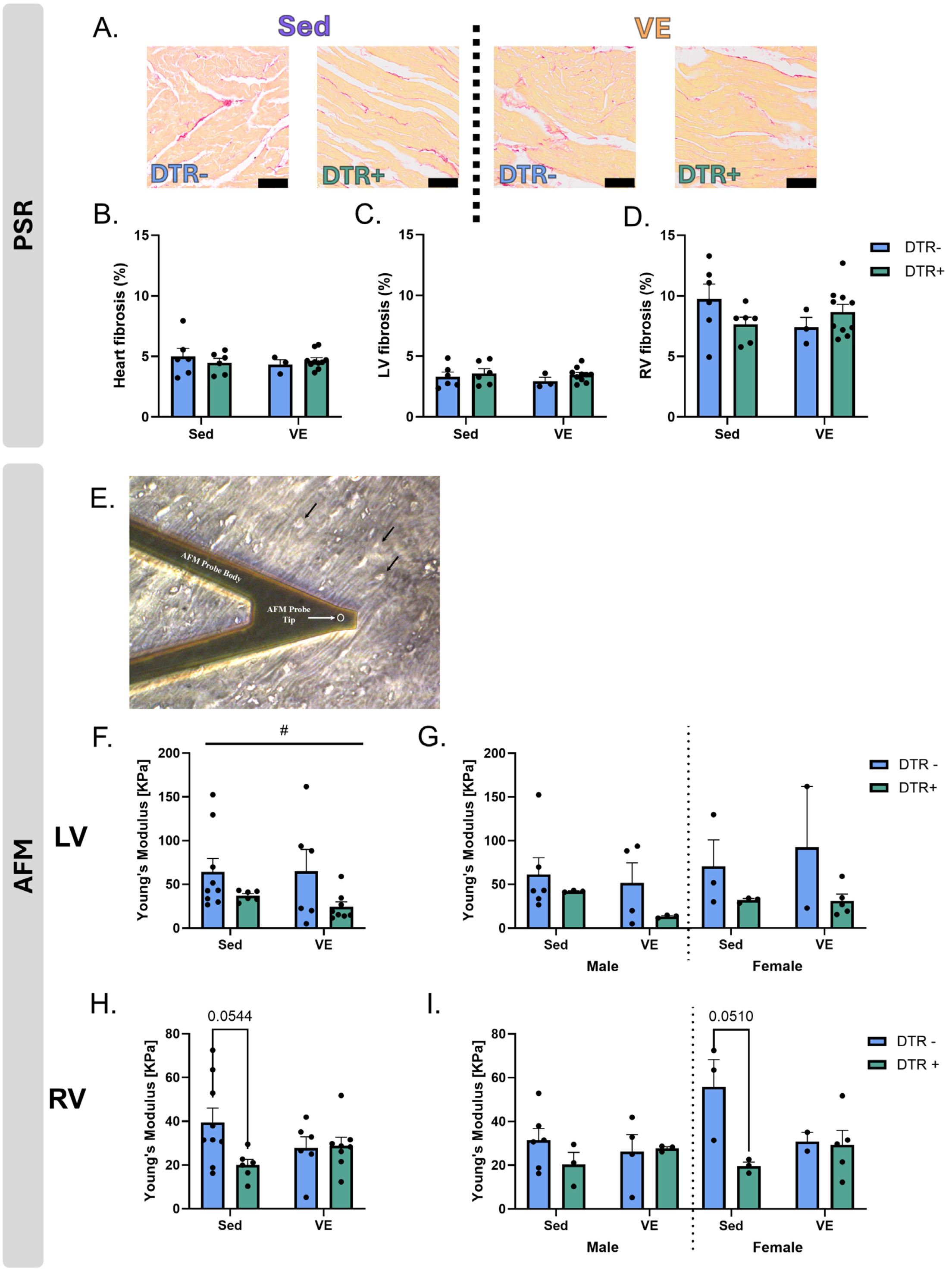

PSR
A.
Sed
VE
DTR-
DTR+
DTR-
DTR+
B.
Heart fibrosis (%)
C.
LV fibrosis (%)
D.
RV fibrosis (%)
DTR-
DTR+
Sed
VE
AFM
E.
AFM Probe Body
AFM Probe Tip
F.
LV
#
Young's Modulus [KPa]
G.
DTR -
DTR+
Male
Female
H.
RV
0.0544
I.
0.0510
DTR -
DTR +

Supplementary Figure 10. Voluntary exercise did not promote pathologic cardiac gene expression. Fresh frozen tissue from DTR^{-} sedentary and VE Cohort 2 and Cohort 3 were analyzed through qPCR. mRNA fold change expression of male **(A)** *Myh6*, **(B)** *Myh7*, **(E)** *ANP*, and **(F)** *BNP*. mRNA fold change expression female **(C)** *Myh6*, **(D)** *Myh7*, **(G)** *ANP*, and **(H)** *BNP* mRNA fold expression. Male n = 3-5 / group. Female n = 3 / group. Myosin heavy chain 6 (alpha) = Myh6. Myosin heavy chain 7 (beta) = Myh7. Atrial natriuretic peptide = ANP. B-type natriuretic peptide = BNP. Abbreviations as in Figure 1 and Figure 3.

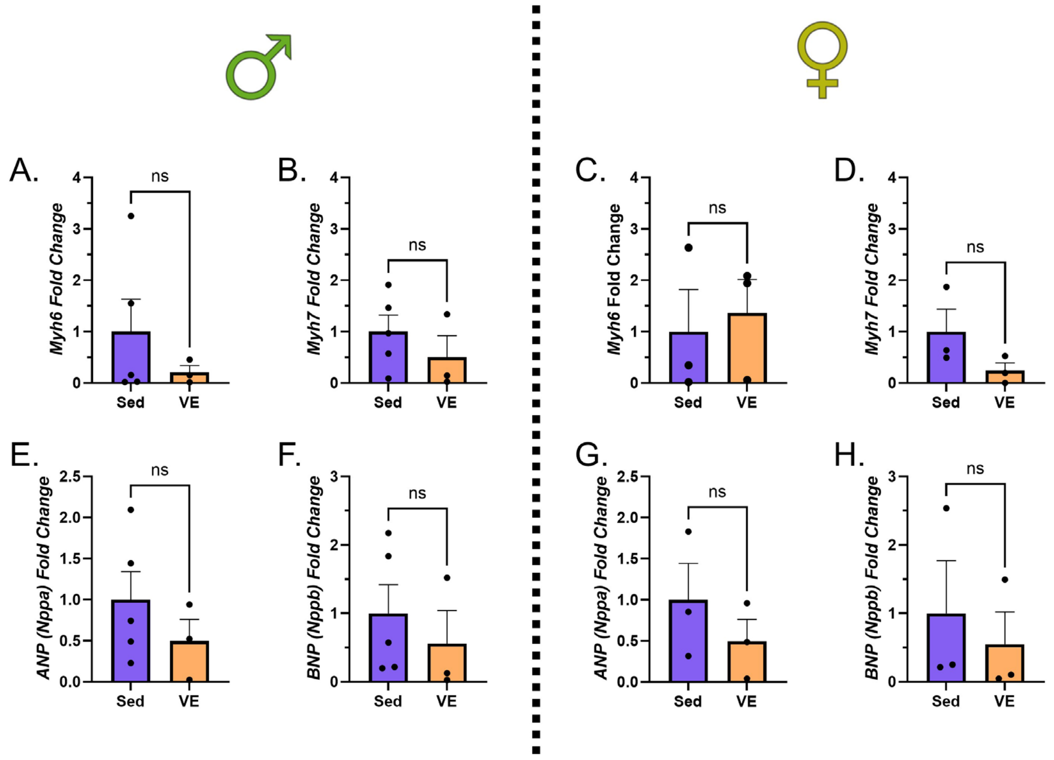

Supplementary Figure 11. Diagram of cardiomyocyte cross-sectional analysis. Cross-sectional WGA (green) cardiomyocyte areas of 250 µm x 250 µm (cyan) were determined and numbered. A random number generator was used to select the areas for analysis to reduce bias. Box # 8 is a representative hand-traced (yellow) example of a 250 µm x 250 µm area. Scale bar = 500 µm

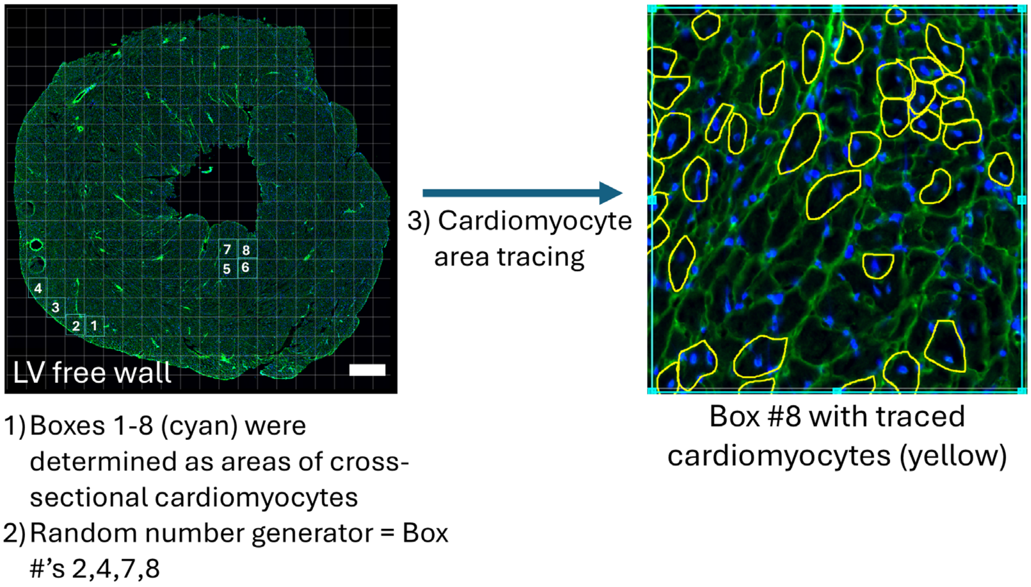

Supplement: Supplementary file 1 [file DataSheet1.pdf]
